# Frailty measurement, prevalence, incidence, and clinical implications in people with diabetes: a systematic review and study-level meta-analysis

**DOI:** 10.1016/S2666-7568(20)30014-3

**Published:** 2020-12

**Authors:** Peter Hanlon, Isabella Fauré, Neave Corcoran, Elaine Butterly, Jim Lewsey, David McAllister, Frances S Mair

**Affiliations:** a General Practice and Primary Care, Institute of Health and Wellbeing, University of Glasgow, Glasgow, UK; b Public Health, Institute of Health and Wellbeing, University of Glasgow, Glasgow, UK; c Health Economics and Health Technology Assessment, Institute of Health and Wellbeing, University of Glasgow, Glasgow, UK

## Abstract

**Background:**

Frailty, a state of increased vulnerability to adverse health outcomes, is important in diabetes management. We aimed to quantify the prevalence of frailty in people with diabetes, and to summarise the association between frailty and generic outcomes (eg, mortality) and diabetes-specific outcomes (eg, hypoglycaemia).

**Methods:**

In this systematic review and study-level meta-analysis, we searched MEDLINE, Embase, and Web of Science for observational studies published between Jan 1, 2001 (the year of the original publication of the Fried frailty phenotype), to Nov 26, 2019. We included studies that assessed and quantified frailty in adults with diabetes, aged 18 years and older; and excluded conference abstracts, grey literature, and studies not published in English. Data from eligible studies were extracted using a piloted data extraction form. Our primary outcome was the prevalence of frailty in people with diabetes. Secondary outcomes were incidence of frailty and generic and diabetes-specific outcomes. Data were assessed by random-effects meta-analysis where possible and by narrative synthesis where populations were too heterogeneous to allow meta-analysis. This study is registered with PROSPERO, CRD42020163109.

**Findings:**

Of the 3038 studies we identified, 118 studies using 20 different frailty measures were eligible for inclusion (n=1 375 373). The most commonly used measures of frailty were the frailty phenotype (69 [58%] of 118 studies), frailty (16 [14%]), and FRAIL scale (10 [8%]). Studies were heterogenous in setting (88 studies were community-based, 18 were outpatient-based, ten were inpatient-based, and two were based in residential care facilities), demographics, and inclusion criteria; therefore, we could not do a meta-analysis for the primary outcome and instead summarised prevalence data using a narrative synthesis. Median community frailty prevalence using frailty phenotype was 13% (IQR 9–21). Frailty was consistently associated with mortality in 13 (93%) of 14 studies assessing this outcome (pooled hazard ratio 1·51 [95% CI 1·30–1·76]), with hospital admission in seven (100%) of seven, and with disability in five (100%) of five studies. Frailty was associated with hypoglycaemia events in one study (<1%), microvascular and macrovascular complications in nine (82%) of 11 studies assessing complications, lower quality of life in three (100%) of three studies assessing quality of life, and cognitive impairment in three (100%) of three studies assessing cognitive impairment. 13 (11%) of 118 studies assessed glycated haemoglobin finding no consistent relationship with frailty.

**Interpretation:**

The identification and assessment of frailty should become a routine aspect of diabetes care. The relationship between frailty and glycaemia, and the effect of frailty in specific groups (eg, middle-aged [aged <65 years] people and people in low-income and lower-middle-income countries) needs to be better understood to enable diabetes guidelines to be tailored to individuals with frailty.

**Funding:**

Medical Research Council.

## Introduction

Clinicians and health-care systems worldwide are facing the challenges associated with ageing populations. Diabetes (type 1 and type 2) is prevalent in up to 30% of people older than 65 years.^1^ Frailty is a key concept for health care, particularly as people age.^2^ Frailty describes a dynamic state of increased vulnerability to adverse health outcomes resulting from loss of physiological reserve.^2^ The prevalence of frailty increases with increasing age.^2^ However, frailty is not universal among older people (aged >65 years), and can also be identified in younger people (aged <65 years), particularly in the context of long-term conditions, including diabetes.^3^, ^4^

The importance of frailty is increasingly recognised in clinical guidelines for diabetes management.^5^, ^6^ Specifically, more relaxed glycated haemoglobin (HbA_1c_) targets are recommended among people who are older or frail.^6^ These recommendations are based on lower life expectancy and greater risks of hypoglycaemia in older or frail individuals.^5^ However, guidelines are not explicit about to whom these recommendations should be applied. Frailty is not a single homogeneous concept, and there is no single standard definition or measure.^2^ Instead, multiple operational definitions of frailty exist.^7^ Some are based on characteristics which are measured directly (frailty measures based on physical assessments such as grip strength and walking pace) or self-reported measures, and others on past medical records. Definitions also vary in their inclusion of cognitive status, social vulnerability, and functional disability.^8^ Differences in the definition and identification of frailty can alter the clinical implications for management.^9^

**Research in context**

**Evidence before this study**

We searched MEDLINE, Embase, and Web of Science from Jan 1, 2001, to Nov 26, 2019, for observational studies published in English that assessed frailty in diabetes (type 1, type 2, or unspecified) using the terms “diabetes” and associated terms and “frail”. We included studies using any frailty measure and done in any setting. We did not identify any existing systematic reviews that synthesise data on the prevalence of frailty in people with diabetes. One review (eight studies) showed increased risks of mortality and cardiovascular events in people with diabetes and frailty, but did not distinguish between different definitions of frailty, nor did it consider other clinical outcomes.

**Added value of this study**

This study shows that frailty is common in diabetes. However, the methods used to identify and define frailty are highly variable between studies. Within the same population, some definitions (eg, frailty index) identify a higher proportion of people as frail than do others (eg, frailty phenotype). Despite this variation in measurement, frailty is consistently associated with a range of adverse outcomes, including mortality, hospital admission, disability, and lower quality of life. Important evidence gaps remain. Frailty is present in middle-aged (aged <65 years) and older people (>65 years) with diabetes; however, variation in prognosis or association with outcomes at different ages has not been widely explored. Evidence from lower-income and lower-middle-income countries is scarce, which is an important gap because of the rising prevalence of diabetes, along with an increasing proportion of older people, in many countries. The absolute risk of mortality associated with frailty is highly variable between studies and frailty definitions. The relationship between glycated haemoglobin (HbA_1c_) and adverse outcomes in frail versus non-frail individuals has not been quantified in the literature, and only one study has assessed the relationship between frailty and hypoglycaemia. These are important research gaps, as clinical guidelines recommend different HbA_1c_ targets in the context of frailty, and lower life expectancy forms part of the rationale for these targets.

**Implications of all the available evidence**

Identifying and assessing frailty should become a routine aspect of diabetes care, which will require frailty screening to become embedded within existing protocols and systems for managing diabetes. There is also a need for a more nuanced understanding of how frailty should be identified and characterised, including the implications of the choice of frailty measure. This is particularly important if clinicians are to identify people likely to benefit from guideline recommendations for managing diabetes in the context of frailty. As these guidelines focus on glycaemic targets, the scarcity of studies exploring the relationship between frailty, HbA_1c_, and clinical outcomes is an important research gap. Because frailty is also prevalent in middle-aged people with diabetes, there is a need to question and explore the clinical implications of frailty across a wider age range, as the basis for current guideline recommendations is based on observations from older populations.

There is, therefore, uncertainty as to how frailty should be identified, measured, and managed, including in the context of diabetes. Because of the complex and multifaceted nature of frailty, understanding its relationship with a broad range of outcomes is important to inform clinical decision making around care and treatment. This systematic review aims to: first, identify frailty measures that have been used to identify frailty in people with diabetes; second, quantify the prevalence of frailty in people with diabetes; and, third, summarise the association between frailty and generic outcomes (eg, mortality), and diabetes-specific clinical outcomes (eg, hypoglycaemia) in the context of diabetes.

## Methods

### Search strategy and selection criteria

We did a systematic review and study-level meta-analysis of observational studies assessing frailty in the context of diabetes. Methods were prespecified and reported according to Preferred Reporting Items for Systematic Reviews and Meta-Analyses (PRISMA) guidelines. Criteria for inclusion are described in detail in the review protocol,^10^ and were deliberately broad in terms of setting, frailty definition, and outcomes. We included studies done in any setting (community, outpatient, inpatient, and residential care). Criteria included observational studies, including cross-sectional and cohort studies, that included adults (≥18 years) with diabetes (any type or unspecified) and quantified frailty in participants with diabetes, using any frailty measure or definition to allow comparison between different methods of identifying frailty. Exclusion criteria were grey literature, conference abstracts and any studies not published in English.

We searched MEDLINE, Embase, and Web of Science databases between Jan 1, 2001 (which was the year of the original publication of the Fried frailty phenotype),^11^ to Nov 26, 2019, using keywords and Medical Subject Headings. The search structure was “diabetes” and “frail” (full search strategy in appendix p 3). We screened all titles and abstracts, and assessed full texts of all relevant articles for eligibility. We supplemented electronic searches by hand-searching reference lists of relevant articles and forward-citation searching using Web of Science. All stages of screening, data extraction, and quality assessment were done independently by two authors (PH and IF, NC, or EB). Discrepancies were resolved by consensus and by a third author (NC or EB).

### Data analysis

Data from eligible studies were extracted using a piloted data extraction form. Differences in data extraction between reviewers were resolved by consensus. We extracted data for study aims, study, design setting, population characteristics (eligibility, recruitment method, summary data for age and sex), diabetes type (type 1, type 2, unspecified), frailty measure (including whether criteria were adapted from the original description of the frailty definition), prevalence of frailty in participants with diabetes, and the association between frailty and clinical outcomes. The risk of bias in the included studies was assessed using an adaptation of the Newcastle-Ottawa tool to make the questions about exposure specific to the assessment of frailty (eg, use of a validated tool) (appendix p 5).^12^

Our primary outcome was prevalence of frailty in people with diabetes. Secondary outcomes were incidence of frailty, generic health-care associated outcomes (including mortality, hospitalisation, health-care utilisation, quality of life, disability, cognitive impairment, and depression), and diabetes-specific outcomes (glycaemic control, macrovascular and microvascular complications).

Estimates of the prevalence of frailty in diabetes are likely to vary depending on the characteristics of the underlying population (eg, age, sex, and ethnicity), definition of diabetes, frailty definition used, adaptations to frailty criteria, and study setting. Because of these multiple sources of heterogeneity, we did a narrative synthesis of prevalence estimates incorporating these features. The quality of the included studies (judged by the quality assessment) was incorporated into the narrative synthesis presented in the text (eg, highlighting where samples were unrepresentative and length of follow-up).

Due to the high likelihood of residual heterogeneity between populations and cohort inclusion criteria, we did not do a meta-analysis of these estimates.^13^

Studies reporting the relationship between frailty and clinical outcomes in diabetes were synthesised using a combination of narrative synthesis and random-effects meta-analysis. A meta-analysis was done only when there were at least two studies assessing the same outcome, using a comparable method of analysis (ie, the same statistical approach was used [eg, Cox proportional hazard model of time to event data for mortality]). Where these studies used the same measure of frailty, a summary estimate was calculated and heterogeneity assessed using *I*^2^ statistic. Where different frailty measures were used to assess the same outcome, studies were grouped by frailty measure and meta-analysed in subgroups (prespecified in the protocol).^10^ Where outcomes or analytic approaches were too heterogeneous, a narrative synthesis was done and data summarised using Harvest plots.^14^ Harvest plots use bars to represent individual studies placed on a matrix to indicate whether the studies showed a positive, negative, or neutral association with the outcome in question, and allow synthesis of heterogeneous outcome data. Data processing and analysis was done using R (version 3.6.1). Meta-analyses were done using RevMan5.

This study is registered with PROSPERO, CRD42020163109.

### Role of the funding source

The funder of the study had no role in study design, data collection, data analysis, data interpretation, or writing of the report. The corresponding author had full access to all the data in the study and had final responsibility for the decision to submit for publication.

## Results

**Figure 1:**
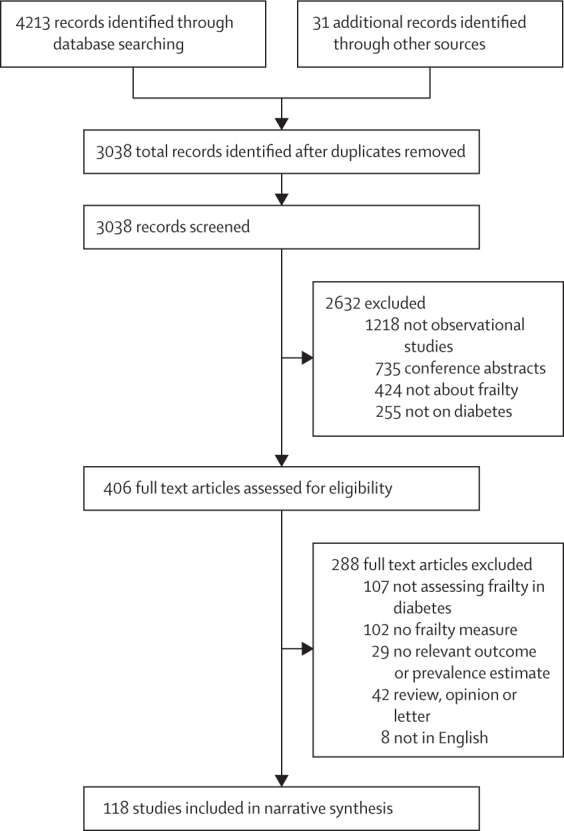
Study selection

After screening 3038 records, we identified 118 (which included 106 cohorts and samples) that met our inclusion criteria (1 375 373 participants overall; figure 1). Details of each included study are summarised in the appendix (pp 12–43).

Most studies were community-based population studies (88 [75%]), 18 (15%) were outpatient studies, ten (8%) were inpatient studies and two (2%) studies were based in residential care facilities. Studies were from a wide range of geographical locations (appendix p 4). Most studies were from high-income (88 [75%] of 118 from 18 countries) or from upper-middle-income countries (27 [23%] from five countries), three studies (3%) were from three lower-middle-income countries and none were from low-income countries. 25 (21%) of 118 studies included people with type 2 diabetes specifically and in 93 (79%) studies the type of diabetes was unspecified. 30 (25%) of 118 studies specifically recruited people with diabetes, while in the remaining 88 (75%) studies, people with diabetes were a subgroup of the study population. Eight (7%) studies assessed specific ethnic groups (one study with African Americans, six studies with three different cohorts of Mexican Americans, and one study with Aboriginal Australians). A wide variety of frailty measures (either validated or well described) were used in the included studies: 20 different measures in total (table). The frailty phenotype was used in 69 (58%) studies; however, in 51 (74%) of these studies the definition of one or more of the five frailty criteria differed from the original description from the Cardiovascular Health Study.^11^ The frailty index (16 [14%] studies) and FRAIL scale (10 [8%] studies) were also used. The remaining 23 studies used other measures of frailty (table).

**Table:** Frailty measures in included studies

	**Components**	**Range and categorisation**	**Included studies (n)**	**Outcomes reported in included studies**
Frailty phenotype*	Five components: unintentional weight loss, exhaustion, low grip strength, slow walking pace, and low physical activity	1–2 criteria: pre-frail; ≥3 criteria: frail	69	Mortality (n=2); HbA_1c_ (n=1); complications† (n=1); cognitive impairment (n=2); disability (n=1); QOL (n=1)
Frailty index*	Count of health-related deficits (≥30, type and number of chosen deficits can vary between studies); total present divided by number of possible deficits	Range 0–1; sometimes categorised (threshold for frailty varies [eg, 0·2, 0·24])	16	Mortality (n=3); hospitalisation (n=1); HbA_1c_ (n=1); complications† (n=1); disability (n=1)
Fatigue, resistance, ambulation, illnesses, & loss of weight scale	Five components (weight loss, fatigue, weakness, ambulation, illness, or comorbidity)	1–2 criteria: pre-frail; ≥3 criteria: frail	10	Mortality (n=4); hospitalisation (n=4); emergency department visit (n=2); disability (n=2); complications† (n=2); depression (n=1)
Clinical frailty scale	Clinical tool based on functional status	Ranges 1 (very fit) to 9 (terminally ill); some dichotomise frail as ≥5	5	Mortality (n=2); HbA_1c_ (n=2); complications† (n=1)
Edmonton frailty scale	Nine components: cognition, general health, functional independence, social support, medication, nutrition, mood, continence, and functional performance	Score 0–17; mild (7–8); moderate (9–10); severe frailty (≥11)	4	Complications† (n=2); depression (n=1); QOL (n=1)
Johns Hopkins adjusted clinical groups	Weighted comorbidity score identified from electronic medical records	Presence of frailty identified by specific indicator conditions	3	HbA_1c_ (n=1); complications† (n=1)
Kihon checklist	Self-administered checklist (components: activities of daily living, exercise, falling, nutrition, oral health, cognition, and depression)	Range 0–25; pre-frail (4–7); frail (≥8)	3	None
Comprehensive geriatric assessment	Multidisciplinary assessment, typically led by a geriatrician, aiming to reach a holistic assessment of health and wellbeing	Frailty identified by clinical judgement rather than predefined criteria	2	Hospitalisation (n=1); hypoglycaemia (n=1); complications† (n=1); depression (n=1); cognitive impairment (n=1); QOL (n=1)
Electronic frailty index	Count of deficits identified from electronic medical records, based on the Frailty index approach	Mild (0·12–0·24); moderate (0·24–0·36); severe frailty (>0·36)	2	HbA_1c_ (n=1); complications† (n=1)
Frailty risk class	List of indicator conditions identified from electronic medical records	Presence of frailty identified by specific indicator conditions	2	Mortality (n=1)
Frailty risk score	Count of 16 frailty risk factors (symptoms, behavioural factors, biomarkers, and nutritional factors)	Range 0–16	1	Mortality (n=1); hospitalisation (n=1); HbA_1c_ (n=1)
Frailty staging system	Seven components (disability, mobility, cognition, vision, hearing, continence, and social support)	Range 0–7; mild (1) moderate (2–3); severe frailty (≥4)	1	Mortality (n=1); cognitive impairment (n=1)
Frailty trait score	12 items across seven components (nutrition, activity, nervous system, vascular system, weakness, endurance, and slowness)	Range 0–49	1	None
Gill index	Composite of chair stand and walking speed tests	Moderate (unable to carry out one element) or severe frailty (both elements)	1	None
Groningen frailty indicator	15 items across four domains (physical, cognitive, social, and psychological)	Range 0–15; ≥4 indicates frailty	1	None
Modified physical performance test	Nine item instrument assessing physical tasks	Range 0–36; moderate (22–29); severe frailty (≤21)	1	Complications† (n=1)
QFrailty	Algorithm based on electronic medical records combining mortality (QMortality score) and hospital admission (QAdmission score) risk	Categorised as mild, moderate, and severe frailty	1	None
RAND-36 questionnaire	Physical function sub-scale of the RAND-36 quesionnaire	Range 0–100; score <80 taken to indicate frailty	1	Mortality (n=1); complications† (n=1)
Study of osteoporotic fracture frailty indicator	Three components (weight loss, chair stand, and exhaustion)	One component: pre-frail; two to three components: frail	1	None‡
Vulnerable elders survey (VES-13)	Telephone questionnaire with 13 components (age, self-rated health, physical function, and disability)	Score ≥4=frail	1	HbA_1c_ (n=1); complications† (n=1)

HbA_1c_=glycated haemoglobin. QOL=quality of life.

* Three studies using these measures assessed more than one measure.

† Diabetes-specific outcomes or complications (ie, microvascular or macrovascular).

‡ These studies considered prevalence of frailty only (ie, they did not assess the association between frailty and any other outcome).

In the 118 included studies, the median number of people with diabetes was 205 (IQR 104–570). Study populations were heterogeneous. Mean age ranged from 50·4 years to 88·0 years (median 72·8 [IQR 69·6–74·4]). Eight (9%) of 88 community-based studies analysed adults of any age. Of these 88 studies, 72 (82%) sampled people above a specified age cutoff (most commonly aged 60 [ten studies] or 65 years [39 studies]). Eight (9%) of 88 studies assessed specific age ranges, with three of these studies including middle-aged people (age ranges 37–73 years, 45–74 years, and 49–65 years). Most community-based studies were judged to be representative in terms of age and sex (determined by sampling methods, response rates and demographics of people included); however, very few reported differences between included participants and non-responders. 14 community-based studies focused on specific populations (ie, four studies on men, two on women, and eight on specific ethnic groups). Whole population studies varied in their sampling method (household survey, postal invitation, stratified sampling, or routine data analysis) and in their exclusion criteria. For example, 81 (69%) of 118 excluded individuals who were institutionalised (eg, living in residential care or nursing homes), people with restricted mobility (unable to attend an assessment), people with cognitive impairment, or people with specific disorders (eg, neurological conditions such as Parkinson's disease or stroke, often when the study included assessments of mobility or functional status). As many of these factors have established associations with frailty, it is likely that variation in these population characteristics will influence the estimated prevalence of frailty in the studies.

**Figure 2:**
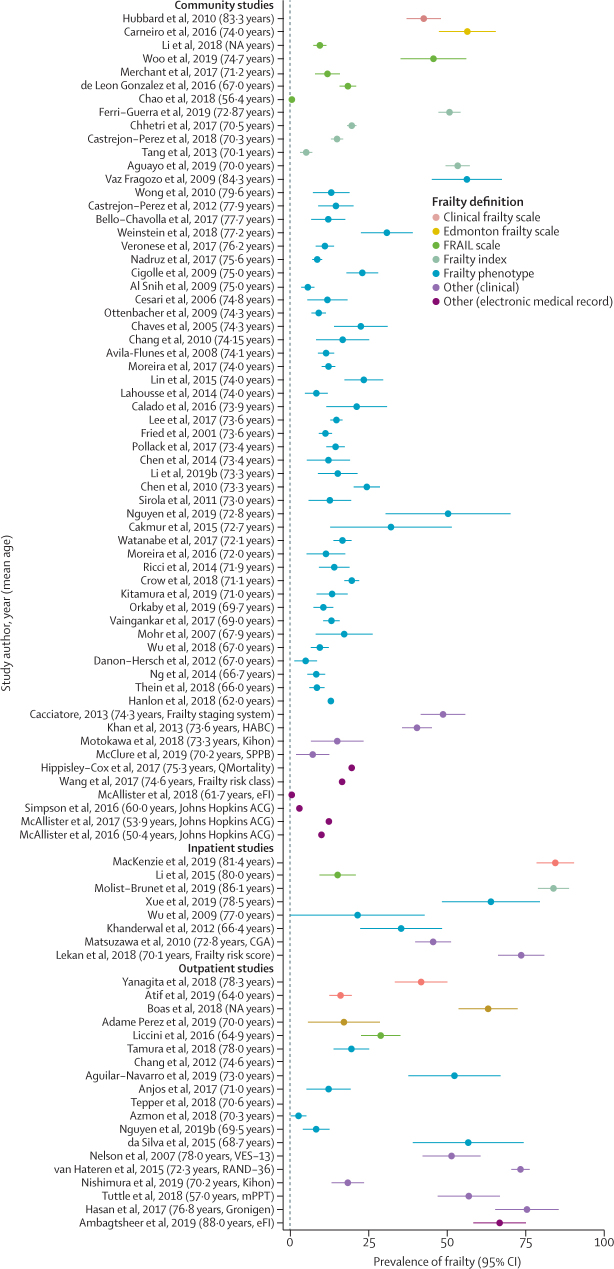
Prevalence of frailty by setting and frailty definition, ordered by mean age of study population Full list of references of all the studies mentioned is included in the appendix (p 46)). eFI=electronic frailty index. ACG=adjusted clinical groups. CGA=comprehensive geriatric assessment. VES-13=vulnerable elders survey RAND-36=research and Development Corporation. Kihon=kihon checklist. mPPT-modified physical performance test. Gronigen=Gronigen frailty indicator.

The prevalence of frailty in people with diabetes is shown in figure 2, with estimates from each study expressed as a proportion (with 95% CIs). Results are stratified by setting and frailty definition and ordered by mean age of the study population. Prevalence estimates varied widely. Median community frailty prevalence using frailty phenotype was 13% (IQR 9–21). Studies with a lower mean age tended to show lower frailty prevalence, particularly those studies without a lower age cutoff. However, prevalence was mixed even among populations with similar mean age and using the same frailty measure (particularly in community-based studies using the frailty phenotype). These differences in results might reflect a combination of differences in the underlying population, variation in exclusion criteria and in methods of recruitment affecting representativeness of the sample, and differences in how frailty components were specified.^15^ Three (3%) of 118 studies used both the frailty index and frailty phenotype.^16^, ^17^, ^18^ In each of these studies, the percentage of people identified as frail was higher using the frailty index (53%, 30%, and 32%)^16^, ^17^, ^18^ compared to using the frailty phenotype (23%, 26%, and 24%),^16^, ^17^, ^18^ highlighting the sensitivity of frailty prevalence to the measure used. Frailty prevalence was also notably high in some ethnic groups (eg, African Americans and Aboriginal Australians) and lower in others (eg, Mexican Americans).^4^, ^19^, ^20^

Diabetes was consistently associated with frailty prevalence after adjustment for age, sex, and other risk factors. Furthermore, diabetes was associated with a greater degree of frailty when assessed using the frailty index.

**Figure 3:**
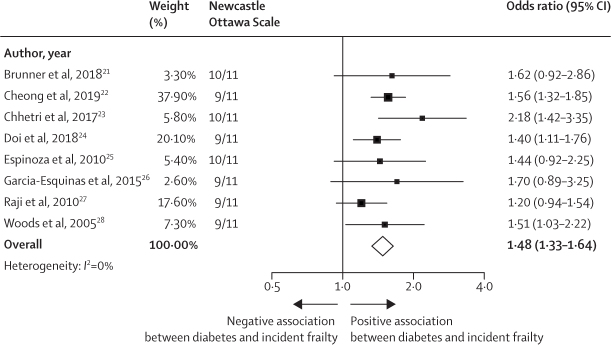
Random-effects meta-analysis of odds of incident frailty associated with diabetes

Eight (9%) of 88 community-based studies assessed the incidence of frailty, all using the frailty phenotype, among people who did not meet criteria for frailty at baseline. In each of these studies, diabetes was included as one of a range of baseline factors associated with the development of frailty. Meta-analysis of these studies shows that diabetes was consistently associated with the development of frailty (pooled odds ratio 1·48 [95% CI 1·33–1·64]; figure 3). Heterogeneity between study estimates was low (*I*^2^=0%) despite variation in the length of follow-up and the variables in each model. The only study^16^ assessing the association between HbA_1c_ and changes in frailty status showed that a higher HbA_1c_ at baseline was associated with worsening frailty over a 10-year period measured using the frailty index. Three studies (3%) of 118 assessed transitions between frailty phenotype states and found that people with diabetes were less likely to improve from a frail to a pre-frail or robust state compared to people without diabetes.^29^, ^30^, ^31^ Together, these data provide evidence that diabetes, and perhaps poor glycaemic control, are risk factors for the development and persistence of frailty.

**Figure 4:**
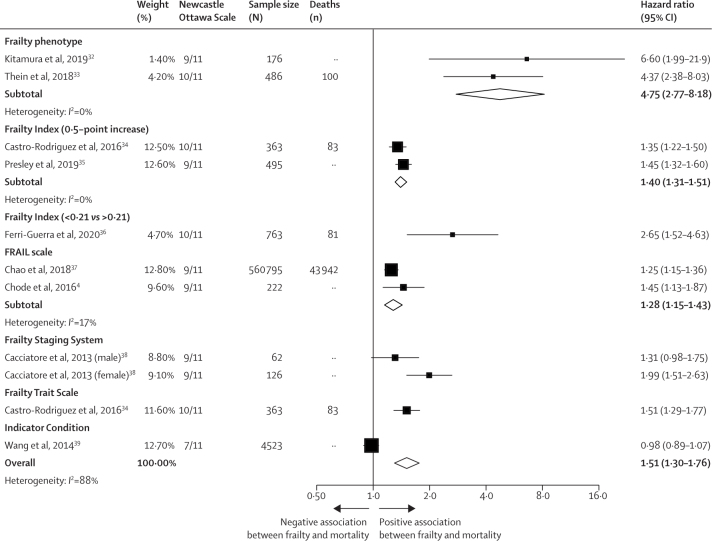
Random effects meta-analysis of association between frailty and mortality

14 (12%) of 118 studies, using eight different frailty measures, assessed the relationship between frailty and all-cause mortality in people with diabetes. Eight of these used time-to-event analyses and were included in a meta-analysis, with each frailty measure as a separate subgroup (figure 4). Frailty was consistently associated with mortality (pooled hazard ratio 1·51 [95% CI 1·30−1·76]); however, the relative effect size varied considerably between studies using different frailty measures (*I*^2^=88% showing high heterogeneity).

Studies varied in length of follow-up, covariate adjustment, and method of mortality assessment, limiting comparison of the absolute mortality rates associated with frailty in diabetes. However, the absolute mortality rate associated with frailty clearly differed between studies. In one study,^40^ hospitalised older patients with diabetes and frailty, according to the Clinical Frailty Scale, had a median life expectancy of 23 months. Mortality incidence in people with frailty was 60 per 1000 person-years in one study using the frailty phenotype in Japan (mean age 72 years),^32^ and 161 per 1000 person-years in another study using the FRAIL scale in Taiwan (mean age 71 years).^37^ Crude mortality rates in three different studies at 10-year follow-up were 50% using the frailty risk class,^38^ 68% using the frailty phenotype,^33^ and 96% using the frailty staging system.^39^

Frailty is therefore consistently associated with all-cause mortality in people with diabetes. However, the method used to assess frailty, along with the underlying population, can lead to wide variation in both the relative and absolute risk of mortality in people identified as frail.

**Figure 5:**
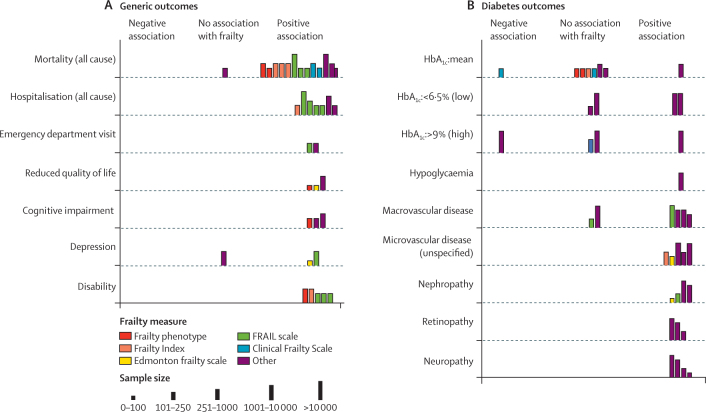
Harvest plot of association between frailty and generic (A) and diabetes-specific (B) clinical outcomes HbA_1c_= glycated haemoglobin.

Studies assessing frailty and health-care utilisation are summarised in figure 5. Details on study methods and effect sizes are presented in the appendix (p 24)). Frailty was consistently associated with increased risk of hospitalisation and with emergency department visits in people with diabetes.

Frailty was consistently associated with disability in five (4%) of 118 studies.^4^, ^33^, ^34^, ^41^, ^42^ Three of these were cross-sectional, while two showed associations between frailty and incident disability over variable lengths of follow-up. Cross-sectional studies also showed associations between frailty and cognitive impairment (three [3%] studies), depression (three [3%] studies), and lower quality of life (three [3%] studies).

The relationship between frailty and diabetes-specific characteristics are shown in figure 5.

Overall, there was little evidence of a relationship between frailty status and mean HbA_1c_. Two of four studies assessing low HbA_1c_ and one of four studies assessing high HbA_1c_ found that people with frailty were more likely to have particularly high or low HbA_1c_ values.^43^, ^44^ Frailty was associated with microvascular and macrovascular complications. These studies were cross-sectional;^36^, ^41^, ^45^, ^46^, ^47^, ^48^, ^49^, ^50^, ^51^, ^52^, ^53^ none assessed changes in HbA_1c_ over time or prospective relationships between frailty and the development of complications. Two studies (2%),^43^, ^44^ which identified frailty using electronic medical records, observed that frail people with overly tight glycaemic control (HbA_1c_ <6·5%) tended to be prescribed hypoglycaemic agents and that these were rarely discontinued despite low HbA_1c_. No included studies assessed the relationship between HbA_1c_ and clinical outcomes in people who were frail. One study assessed the relationship between frailty and hypoglycaemic episodes.^45^ Frailty, identified by multidisciplinary comprehensive geriatric assessment, was associated with a higher incidence of hypoglycaemic episodes, as well as greater risk of hospitalisation with hypoglycaemia. No studies using either epidemiological or clinical measures to identify frailty examined hypoglycaemia as an outcome.

No studies assessed the relationship between frailty and glycaemic variability or the relationship between HbA_1c_ and clinical outcomes in the context of frailty.

## Discussion

This systematic review synthesised data from 118 studies from 18 high-income, five upper-middle-income, and three lower-middle-income countries that assessed the relationship between frailty and diabetes. Frailty was measured using a range of different scales, incorporating different constructs and developed for different purposes. However, across all measures used, frailty was prevalent in community and hospital-based settings and associated with various adverse clinical outcomes, including mortality, hospitalisation, lower quality of life and disability. In community settings, studies showed that frailty prevalence can be expected to lie between 10% and 25% in people with diabetes older than 60 years. Frailty was also present in people younger than 65 years, although this was only examined in six studies. Frailty also appears to be more common in some ethnic groups (eg, Aboriginal Australians and African Americans) although this was only examined in eight studies. Diabetes was also associated with the development and progression of frailty. There were cross-sectional associations between frailty and microvascular and macrovascular complications but not higher mean HbA_1c_. This is notable as clinical guidelines recommend higher HbA_1c_ targets in people with frailty.^5^

Clinicians managing diabetes will encounter frailty regardless of clinical setting. In clinical contexts, a nuanced approach that involves differentiating between levels of frailty and understanding individual patient needs and priorities within the context of frailty is likely to be important, rather than a one-size-fits-all approach to identifying frailty. The identification and assessment of frailty should become part of routine management of people with diabetes. The included studies show that frailty can also be present in younger people with diabetes, including people younger than 65 years. However, the prognostic implications of frailty in diabetes at younger ages have not been examined.

Our findings show that diabetes is a risk factor for the development and progression of frailty. Possible mechanisms include accelerated muscle loss and sarcopenia in diabetes,^54^ along with neuropathic and inflammatory mechanisms,^55^ and shared cardiovascular risk factors.^56^ There is emerging evidence that nutritional and exercise-based interventions can limit the development of frailty in community settings.^57^ However, diabetes was not considered or analysed separately in these studies; therefore, people with diabetes would have been eligible for inclusion, but the findings relate to the population in general and not to diabetes specifically. Non-pharmacological management of diabetes might be synergistic with efforts to prevent the development of frailty. Measuring frailty at baseline and as an outcome in trials of diabetes interventions would be an important step in understanding if and how interventions might mitigate frailty.

The importance of frailty is recognised in several national and international diabetes guidelines.^5^, ^6^, ^58^ Specifically, more relaxed HbA_1c_ targets are recommended, and the risks of hypoglycaemia are highlighted.^6^, ^58^ An international position statement on frailty in diabetes recommended aiming for the tightest control that could be achieved, while minimising the risk of hypoglycaemia.^5^ In mild-to-moderate frailty a target of 7·0–8·0% was recommended and in severe frailty 7·5–8·5% was considered more protective.^5^ This review showed an association between frailty and HbA_1c_ values that were either higher (ie, >9·0%) or lower (ie, <6·5%) than standard targets. Although higher values can be explained by higher targets, the association between frailty and low HbA_1c_ values suggests that many patients with diabetes and frailty might be over-treated. People with frailty and excessively low HbA_1c_ were prescribed hypoglycaemic drugs,^45^ which tended not to be discontinued over time.^43^ Continuing hypoglycaemic agents despite low HbA_1c_ could put people with frailty at greater risk than if these agents were discontinued.

It is also notable that only one study^45^ in this Review quantified the risk of hypoglycaemia in frailty, suggesting that the association between current models of frailty and hypoglycaemia has largely been unquantified. The guideline recommendations are generally based on the high proportion of older people among those presenting with hypoglycaemic complications,^59^, ^60^ as well as data from trials such as ACCORD^61^ in which older patients (>80 years) had particularly high rates of hypoglycaemia when randomly assigned to the intervention groups. Although this provides evidence of the greater risk of hypoglycaemia, particularly in older people, it is not clear if current measures of frailty accurately identify people at greatest risk of hypoglycaemia. Several of the included studies identified frailty in relatively young people with diabetes; however, it is not clear if frailty is associated with similar risks of hypoglycaemia in younger populations. Because the choice of frailty measure, and the way it is implemented has considerable influence over the population that is identified as frail,^15^ it is not clear how best to identify people with diabetes and frailty who are most likely to benefit from these recommendations around HbA_1c_ targets.

Greater consistency in how frailty is measured and reported would improve our understanding of the implications of frailty. However, as frailty is a complex and multifaceted state, broad agreement on a single definition is unlikely.^2^, ^8^ Translation to clinical practice is a key consideration in analysis of frailty because the most frequently used epidemiological measures (such as the Frailty Phenotype) are rarely incorporated into routine health care. The high prevalence and clinical importance of frailty in diabetes are clear, and there is therefore a need to advance our understanding of how frailty in diabetes should be managed. To do so will mean explicitly measuring frailty in diabetes trials and interventions. Such measurement is particularly important as recommendations for the management of diabetes in the context of frailty are based on studies in which frailty was not directly quantified. Because of the variation in how frailty is measured, there is a risk that recommendations will be applied inconsistently, and perhaps inappropriately, in clinical practice. Frailty-specific evidence in the context of diabetes is required to refine the management of people living with frailty.

Our review used a comprehensive search strategy supplemented by hand-searching of relevant literature. However, our search was limited to studies published in English and excluded grey literature and conference abstracts, which could result in language or publication bias. Because the included studies were observational in nature, the relationships between diabetes and frailty cannot be assumed to be causal. There was considerable heterogeneity between included studies, in terms of inclusion criteria and representativeness (introducing potential selection bias), frailty measures (validated *vs* adapted), adaptation of frailty criteria, and study settings. Although we explored the effect on frailty prevalence of some of these factors, we were limited by the reporting of these in the included studies and the variable level of detail provided, particularly around non-response rates and completeness of follow-up data. It was therefore not possible to specify which factors drove the heterogeneity in prevalence estimates.

Frailty identification, assessment, and management should be part of routine diabetes care, which will require integration and embedding of frailty assessment tools into existing templates and protocols. Frailty is not a homogenous entity, and the prognosis and implications of frailty are likely to differ depending on the level of frailty and how it is defined, as well as by other factors such as age. Management must therefore be tailored to the individual. A nuanced and consistent understanding of frailty is needed to inform the evidence base. There is a need to examine the differential consequences of frailty in different sub-populations (such as younger people and people from different ethnic groups). Future research should also focus more on lower-income and middle-income countries, in which diabetes and ageing are growing public health concerns. Finally, despite guidelines calling for lower glycaemic targets in people with diabetes and frailty, HbA_1c_ is below target in many people. Longitudinal assessments of the consequences of glycaemic control in the context of frailty are largely absent from literature. These gaps should be addressed to improve management of people living with diabetes and frailty.
