# Supplementary material for: Frailty measurement, prevalence, incidence, and clinical implications in people with diabetes: a systematic review and study-level meta-analysis
Source: Lancet Healthy Longev. 2020 Dec;1(3):e106–16. doi: 10.1016/S2666-7568(20)30014-3 (PMC7721684; doi:10.1016/S2666-7568(20)30014-3)
Supplement: Supplementary appendix [file mmc1.pdf]

THE LANCET
Healthy Longevity

Supplementary appendix

This appendix formed part of the original submission and has been peer reviewed. We post it as supplied by the authors.

Supplement to: Hanlon P, Fauré I, Corcoran N, et al. Frailty measurement, prevalence, incidence, and clinical implications in people with diabetes: a systematic review and study-level meta-analysis. *Lancet Healthy Longev* 2020; published online Nov 19. https://doi.org/10.1016/S2666-7568(20)30014-3.

Supplementary material

Contents

Medline Search Strategy 3
Search Terms 3
Language restriction 3
Years searched 3
Countries of included studies 4
The Newcastle-Ottawa Scale – Adaptation of criteria 5
1 – Representativeness of the exposed (i.e. frail) cohort 5
2 – Selection of the non-exposed (i.e. non-frail) cohort 5
3 – Ascertainment of exposure 5
4 – Non-respondents 5
5 – Demonstration that outcome of interest was not present at the start of the study 5
Comparability: 6
1 – Comparability of the cohorts on the basis of the design or analysis being controlled for confounders 6
Outcomes: 6
1 – Assessment of outcomes 6
2 – Follow-up long enough for outcomes to occur 6
3 – Adequacy of follow-up of cohorts 6
Quality assessment – Adapted Newcastle Ottawa Scale 7
Details of studies of frailty prevalence 12
Outcomes of included studies 19
Mortality 19
Hospitalisation and Emergency Department Attendance 24
HbA1c 26
Diabetes complications 29
Disability 34
Cognitive impairment 40
Quality of Life 40
Depression 42
Assessment of publication bias 44
References of included studies 46

Medline Search Strategy

Search Terms

1. Exp Frailty/
2. Exp Frail Elderly/
3. Frail*.tw
4. 1 or 2 or 3
5. Exp Diabetes Mellitus
6. Diabet*.tw
7. (IDDM or NIDDM or MODY or T1DM, or T2DM or T1D or T2D).tw
8. (non insulin* depend* or non insulin depend* or non insulin?depend* or non insulin ?depend).tw
9. (insulin* depend* or insulin ?depend*).tw
10. 5 or 6 or 7 or 8 or 9
11. Exp Diabetes Insipidus/
12. Diabet* insipidus.tw
13. 11 or 12
14. 10 not 13
15. 4 and 14

Language restriction

None applied to search (non-English language studies excluded at screening stage)

Years searched

2001-November 2019

Countries of included studies

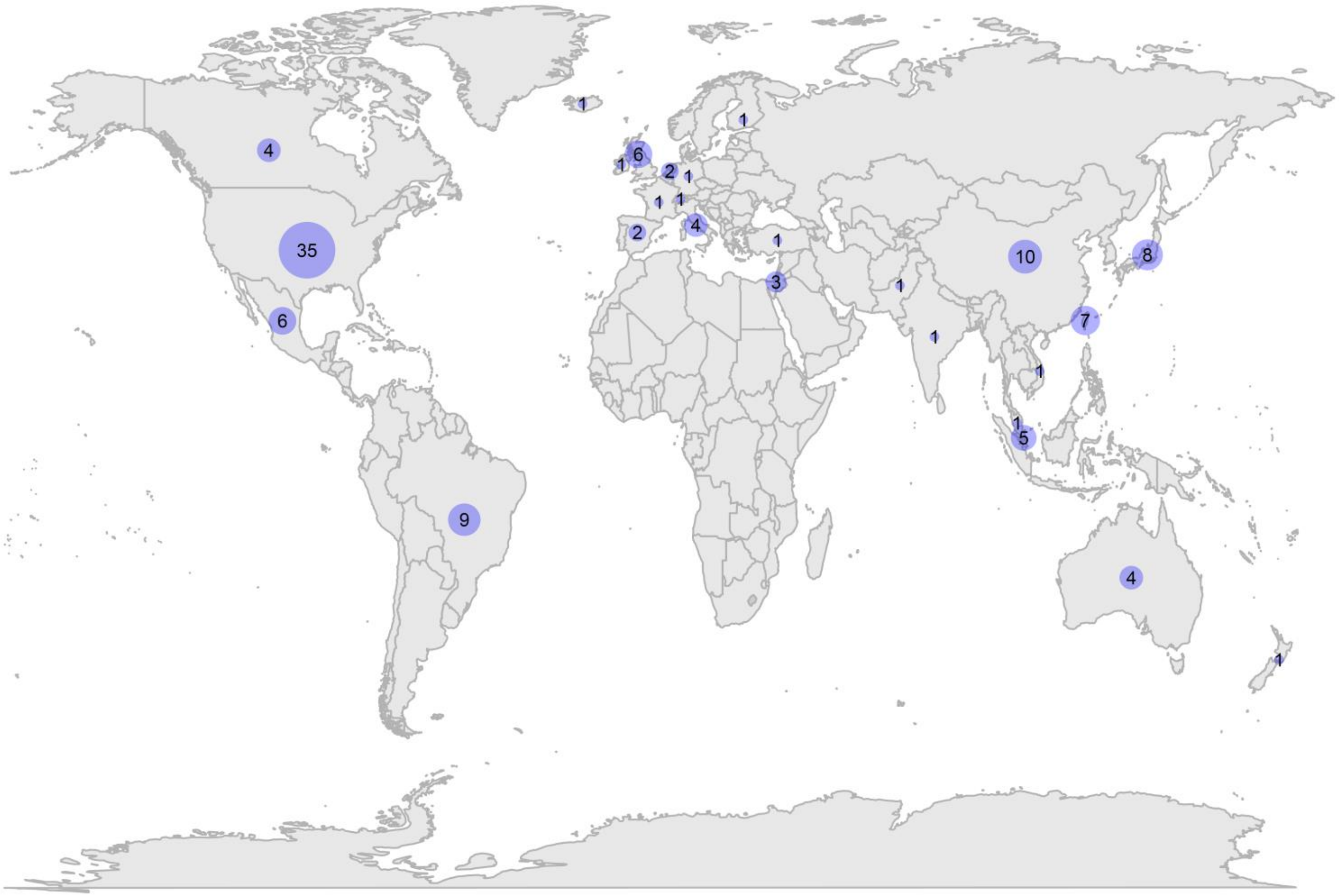

The Newcastle-Ottawa Scale – Adaptation of criteria

Adaptation for studies assessing the prevalence and impact of frailty in diabetes

1 – Representativeness of the exposed (i.e. frail) cohort

a) Truly representative (one star)

b) Somewhat representative (one star)

c) Selected group

d) No description of the derivation of the cohort

2 – Selection of the non-exposed (i.e. non-frail) cohort

a) Drawn from the same community as the exposed cohort (one star)

b) Drawn from a different source

c) No description of the derivation of the non-exposed cohort

3 – Ascertainment of exposure (adapted for measurement of frailty)

a) Validated measurement tool for frailty (two stars)

b) Non-validated measurement tool, but the tool is available or described (one star)

c) No description of measurement tool

4 – Non-respondents

a) Comparability between respondents and non-respondents' characteristics is established, and the response rate is satisfactory (one star)
b) The response rate is unsatisfactory, or the comparability between respondents and non-respondents is unsatisfactory

c) No description of the response rate of the characteristics of the responders and non-responders

5 – Demonstration that outcome of interest was not present at the start of the study

a) Yes (one star)

b) No

Comparability:

1 – Comparability of the cohorts on the basis of the design or analysis being controlled for confounders

a) The study controls for age and sex (one star)

b) The study controls for other factors (one star)

c) Cohorts are not comparable on the basis of the design or analysis controlled for confounders

Outcomes:

1 – Assessment of outcomes

a) Independent assessment (one star)

b) Record linkage (one star)

c) Self-report

d) No description

e) Other

2 – Follow-up long enough for outcomes to occur

a) Yes (one star)

b) No

3 – Adequacy of follow-up of cohorts

a) Complete follow-up: all subjects accounted for (one star)

b) Subjects lost to follow-up unlikely to introduce bias – number lost less than or equal to 20% or description of those lost suggested no different from those followed (one star)

c) Follow-up rate less than 80% and no description of those lost

d) No statement

Quality assessment – Adapted Newcastle Ottawa Scale

Author	Year	Representative	Selection of non frail comparison	Ascertainment of exposure frailty	Non respondents	Outcome not present at start	Controls for age and sex	Control for other factors	Outcome assessment	Length of follow up	Adequacy of follow up	Cross-sectional score	Longitudinal score
Adame Perez	2019	0	1	2	0	-	-	-	-	-	-	3/5	-
Aguilar-Navarro	2019	0	1	1	0	-	-	-	-	-	-	2/5	-
Aguayo	2019	1	1	2	0	-	-	-	-	-	-	4/5	-
Al Snih	2009	1	1	1	0	-	-	-	-	-	-	3/5	-
Almeida	2016	1	1	2	0	-	-	-	-	-	-	4/5	-
Ambagtsheer	2019	0	1	1	0	-	-	-	-	-	-	2/5	-
Anjos	2017	0	1	1	0	-	-	-	-	-	-	2/5	-
Atif	2019	0	1	2	1	-	-	-	-	-	-	4/5	-
Avila_flunes	2008	1	1	1	0	-	-	-	-	-	-	3/5	-
Azmon	2018	0	1	2	0	-	-	-	-	-	-	3/5	-
Bello-Chavolla	2017	1	1	1	0	-	-	-	-	-	-	3/5	-
Boas	2018	0	1	2	0	-	-	-	-	-	-	3/5	-
Bouillon	2013	1	1	2	0	-	-	-	-	-	-	4/5	-
Brunner	2018	1	1	2	0	1	1	1	1	1	1	4/5	10/11
Cacciatore	2013	1	1	1	0	1	1	1	1	1	1	3/5	9/11
Cakmur	2015	1	1	1	0	-	-	-	-	-	-	3/5	-
Calado	2016	1	1	1	0	-	-	-	-	-	-	3/5	-
Carneiro	2016	1	1	2	0	-	-	-	-	-	-	4/5	-
Castrejon-Perez	2018	1	1	2	0	-	-	-	-	-	-	4/5	-

Castrejon-Perez	2012	1	1	1	0	-	-	-	-	-	-	3/5	-
Castrejon-Perez	2017	1	1	2	0	-	-	-	-	-	-	4/5	-
Castro-Rodriguez	2016	1	1	2	0	1	1	1	1	1	1	4/5	10/11
Cesari	2006	1	1	1	0	-	-	-	-	-	-	3/5	-
Chang	2010	1	1	1	0	-	-	-	-	-	-	3/5	-
Chang	2012	0	1	1	0	-	-	-	-	-	-	2/5	-
Chao	2018	1	1	1	0	1	1	1	1	1	1	3/5	9/11
Chaves	2005	1	1	2	0	-	-	-	-	-	-	4/5	-
Chen	2010	1	1	2	0	-	-	-	-	-	-	4/5	-
Chen	2014	1	1	2	0	-	-	-	-	-	-	4/5	-
Cheong	2020	1	1	1	0	1	1	1	1	1	1	3/5	9/11
Chhetri	2017	1	1	2	0	1	1	1	1	1	1	4/5	10/11
Chiu	2019	1	1	1	1	1	1	1	1	1	1	4/5	10/11
Chode	2016	1	1	2	0	1	1	1	1	1	0	4/5	9/11
Cigolle	2009	1	1	2	1	-	-	-	-	-	-	5/5	-
Crow	2018	1	1	1	0	-	-	-	-	-	-	3/5	-
da Silva	2015	0	1	1	0	-	-	-	-	-	-	2/5	-
Danon-Hersch	2012	1	1	1	0	-	-	-	-	-	-	3/5	-
de Leon Gonzalez	2016	1	1	2	0	-	-	-	-	-	-	4/5	-
Doi	2018	1	1	1	0	1	1	1	1	1	1	3/5	9/11
Espinoza	2010	1	1	2	0	1	1	1	1	1	1	4/5	10/11
Espinoza	2012	1	1	2	1	1	1	1	1	1	1	5/5	11/11
Espinoza	2015	1	1	2	0	-	-	-	-	-	-	4/5	-
Ferri-Guerra	2019	1	1	2	0	1	1	1	1	1	1	4/5	10/11
Fried	2001	1	1	2	0	-	-	-	-	-	-	4/5	-
Garcia-Esquinas	2015	1	1	1	0	1	1	1	1	1	1	3/5	9/11
Hanlon	2018	0	1	1	0	-	-	-	-	-	-	2/5	-

Hasan	2017	0	1	2	0	-	-	-	-	-	-	3/5	-
Hippisley-Cox	2017	1	1	1	0	-	-	-	-	-	-	3/5	-
Howrey	2018	1	1	1	0	-	-	-	-	-	-	3/5	-
Hubbard	2010	0	1	2	0	1	0	0	1	1	1	3/5	7/11
Hyde	2019	1	1	2	0	-	-	-	-	-	-	4/5	-
Khan	2013	1	1	1	0	-	-	-	-	-	-	3/5	-
Khanderwal	2012	0	1	1	0	-	-	-	-	-	-	2/5	-
Kirkwood	2019	1	1	1	0	-	-	-	-	-	-	3/5	-
Kitamura	2019	1	1	1	0	1	1	1	1	1	1	3/5	9/11
Lahousse	2014	1	1	1	0	-	-	-	-	-	-	3/5	-
Lee	2017	1	1	2	0	-	-	-	-	-	-	4/5	-
Lee	2011	1	1	1	0	-	-	-	-	-	-	3/5	-
Lee	2014	1	1	1	0	1	1	1	1	1	1	3/5	9/11
Lekan	2018	0	1	1	0	1	1	0	1	0	1	2/5	6/11
Li	2018	1	1	1	0	0	1	1	0	0	1	3/5	6/11
Li	2019	1	1	2	0	-	-	-	-	-	-	4/5	-
Li	2019b	1	1	1	0	-	-	-	-	-	-	3/5	-
Li	2015	0	1	2	0	1	1	1	1	1	0	3/5	8/11
Li	2016	0	1	2	0	-	-	-	-	-	-	3/5	-
Liccini	2016	0	1	2	0	1	1	1	1	1	0	3/5	8/11
Lin	2015	1	1	1	0	-	-	-	-	-	-	3/5	-
MacKenzie	2019	0	1	2	0	-	-	-	-	-	-	3/5	-
Matsuzawa	2010	0	1	1	0	-	-	-	-	-	-	2/5	-
McAllister	2018	1	1	2	0	1	1	1	1	1	1	4/5	10/11
McAllister	2016	1	1	1	0	-	-	-	-	-	-	3/5	-
McAllister	2017	1	1	1	0	1	1	1	1	1	1	3/5	9/11
McClure	2019	1	1	1	0	-	-	-	-	-	-	3/5	-
Merchant	2017	1	1	2	0	-	-	-	-	-	-	4/5	-
Mohr	2007	1	1	1	0	-	-	-	-	-	-	3/5	-

Molist-Brunet	2019	0	1	1	0	-	-	-	-	-	-	2/5	-
Moreira	2017	1	1	2	0	-	-	-	-	-	-	4/5	-
Moreira	2016	1	1	1	0	-	-	-	-	-	-	3/5	-
Motokawa	2018	1	1	2	0	-	-	-	-	-	-	4/5	-
Nadruz	2017	1	1	1	0	-	-	-	-	-	-	3/5	-
Nelson	2007	0	1	1	0	-	-	-	-	-	-	2/5	-
Ng	2014	1	1	1	0	-	-	-	-	-	-	3/5	-
Nguyen	2019	1	1	1	0	-	-	-	-	-	-	3/5	-
Nguyen	2019b	0	1	1	0	-	-	-	-	-	-	2/5	-
Nishimura	2019	0	1	2	0	-	-	-	-	-	-	3/5	-
Orkaby	2019	1	1	2	0	-	-	-	-	-	-	4/5	-
Ottenbacher	2009	1	1	1	0	-	-	-	-	-	-	3/5	-
Pilotto	2014	0	1	1	0	1	1	1	1	1	0	2/5	7/11
Pollack	2017	1	1	1	0	1	1	1	1	1	1	3/5	9/11
Presley	2019	0	1	2	0	1	1	1	1	1	1	3/5	9/11
Raji	2010	1	1	1	0	1	1	1	1	1	1	3/5	9/11
Ricci	2014	1	1	1	0	-	-	-	-	-	-	3/5	-
Saum	2012	1	1	1	0	-	-	-	-	-	-	3/5	-
Simpson	2016	1	1	1	0	1	1	1	1	1	1	3/5	9/11
Sirola	2011	1	1	1	0	-	-	-	-	-	-	3/5	-
Tamura	2018	0	1	1	0	-	-	-	-	-	-	2/5	-
Tang	2013	1	1	2	0	-	-	-	-	-	-	4/5	-
Tepper	2018	0	1	1	0	-	-	-	-	-	-	2/5	-
Thein	2018	1	1	2	0	1	1	1	1	1	1	4/5	10/11
Tuttle	2018	0	1	1	0	-	-	-	-	-	-	2/5	-
Vaingankar	2017	1	1	1	0	-	-	-	-	-	-	3/5	-
van Hateren	2015	0	1	1	0	-	-	-	-	-	-	2/5	-
Vaz Fragozo	2009	1	1	1	0	-	-	-	-	-	-	3/5	-
Veronese	2017	1	1	1	0	-	-	-	-	-	-	3/5	-

Veronese	2016	1	1	1	0	-	-	-	-	-	-	3/5	-
Wang	2014	1	1	1	0	-	-	-	-	-	-	3/5	-
Wang	2017	1	1	1	0	1	1	1	1	0	0	3/5	7/11
Wata-be	2017	1	1	1	0	-	-	-	-	-	-	3/5	-
Weinstein	2018	1	1	1	0	-	-	-	-	-	-	3/5	-
Wong	2018	1	1	2	0	-	-	-	-	-	-	4/5	-
Wong	2010	1	1	1	0	-	-	-	-	-	-	3/5	-
Woo	2019	1	1	1	0	-	-	-	-	-	-	3/5	-
Woods	2005	1	1	1	0	1	1	1	1	1	1	3/5	9/11
Wu	2009	0	1	1	0	-	-	-	-	-	-	2/5	-
Wu	2018	1	1	1	0	-	-	-	-	-	-	3/5	-
Xue	2019	0	1	1	0	-	-	-	-	-	-	2/5	-
Ya-gita	2018	0	1	2	0	-	-	-	-	-	-	3/5	-
Zaslavsky	2016	1	1	1	0	-	-	-	-	-	-	3/5	-

Details of studies of frailty prevalence

Author, Year	Country	Cohort	Setting	Frailty measure	Lower age limit or specified range	Type of diabetes	Number of people with diabetes	Mean/median age*	Number (%) female
Adame Perez 2019	Canada		outpatient	Edmonton	≥65	unspecified	41	70 (65-74)	15 (36.6%)
Aguilar-Navarro 2019	Mexico	Recruited from memory clinic	outpatient	Fried	≥60	unspecified	44	73 (6.6)	NA
Aguayo 2019	UK	English Longitudinal Study of Aging	community	FI	≥50	unspecified	635	70 (65-77)	2995 (55.7%)
Al Snih 2009	USA	H-EPESE study	community	Fried	≥67	unspecified	431	75 (6)	NA
Ambagtsheer 2019	Australia	Database of 10 aged care facilities	residential_care	eFI	≥75	unspecified	120	88 (9)	394 (66.6%)
Anjos 2017	Brazil	Community diabetes clinic	outpatient	Fried	≥65	Type 2	82	71 (4.8)	82 (100%)
Atif 2019	Pakistan	Two diabetes outpatient clinics	outpatient	CFS	≥60	Type 2	400	64 (5.5)	215 (53.8%)
Avila_flunes 2008	France	Three-City study	community	Fried	≥65	unspecified	565	74.1 (5.2)	3726 (61.3%)
Azmon 2018	Israel	Specialist diabetes outpatient service	outpatient	Fried	≥60	Type 2	153	70.3	NA
Bello-Chavolla 2017	Mexico	Coyoacán Cohort Study	community	Fried	≥70	Type 2	135	77.7 (5.8)	NA
Boas 2018	Brazil		outpatient	Edmonton	≥60	unspecified	100	NA	126 (84%)

Cacciatore 2013	Italy	Osservatorio Geriatrico Regione Campania	community	Frailty staging system	≥65	unspecified	188	74.3 (6.4)	712 (55.3%)
Cakmur 2015	Turkey		community	Fried	≥65	unspecified	22	72.7 (7.7)	90 (53.6%)
Calado 2016	Brazil	FIBRA study	community	Fried	≥65	unspecified	67	73.9 (6.5)	249 (64.7%)
Carneiro 2016	Brazil		community	Edmonton	≥60	unspecified	114	74 (7.14)	327 (64%)
Castrejon-Perez 2018	Mexico	ENSANUT	community	FI	≥60	unspecified	1236	70.3 (7.8)	2943 (54.7%)
Castrejon-Perez 2012	Mexico	Mexican Study of Nutritional and Psychosocial Markers of Frailty (the Coyoacan cohort)	community	Fried	≥70	unspecified	147	77.9 (6.3)	NA
Cesari 2006	Italy	In Chianti study	community	Fried	≥65	unspecified	95	74.8 (6.8)	NA
Chang 2010	USA	WHAS I and II	community	Fried	70-79	unspecified	73	74.15 (2.8)	NA
Chang 2012	Taiwan		outpatient	Fried	≥65	unspecified	35	74.6 (6.3)	NA
Chao 2018	Taiwan	Longitudinal Cohort of Diabetes Patients database	community	FRAIL	≥20	Type 2	560795	56.4 (13.8)	258526 (46.1%)
Chaves 2005	USA	WHAS I and II	community	Fried	70-80	unspecified	90	74.3 (2.9)	NA
Chen 2010	Taiwan	Survey of Health and Living Status of the Elderly in Taiwan	community	Fried	≥65	unspecified	398	73.3 (1.5)	NA
Chen 2014	Taiwan	The Coming of the Aging Society:<br><br>An Integrative Study on Social	community	Fried	≥65	unspecified	84	73.4	239 (48.3%)

		Planning in Taiwan in 2025							
Chhetri 2017	China	BLSA-II	community	FI	≥55	unspecified	2634	70.5 (7.8)	NA
Cigolle 2009	USA	HRS	community	Fried	≥65	unspecified	260	75	NA
Crow 2018	USA	National Health and Nutrition Examination Survey	community	Fried	≥60	unspecified	1060	71.1 (0.19)	NA
da Silva 2015	Brazil		outpatient	Fried	60-79	Type 2	30	68.7 (6.92)	NA
Danon-Hersch 2012	Switzerland	Lc65+	community	Fried	65-70	unspecified	129	67 (65-70)	515 (40.1%)
de Leon Gonzalez 2016	Mexico	Mexican Health and Aging Study	community	FRAIL	≥60	unspecified	801	67	NA
Ferri-Guerra 2019	USA		community	FI	≥65	unspecified	763	72.87 (6.78)	13 (1.7%)
Fried 2001	USA	CHS	community	Fried	≥65	unspecified	840	73.6	3079 (57.9%)
Hanlon 2018	UK	UK Biobank	community	Fried	40-70	unspecified	24696	62	NA
Hasan 2017	Malaysia		residential_care	Gronigen	≥65	unspecified	69	76.8 (7.8)	126 (62.4%)
Hippisley-Cox 2017	UK	Qresearch database	community	Qmortality	≥65	Type 2	73909	75.3 (8)	274931 (55%)
Hubbard 2010	England	CSHA	community	CFS	≥70	unspecified	310	83.3	NA
Khan 2013	USA	Health ABC study	community	HABC	70-79	unspecified	404	73.6 (2.9)	1472 (52.1%)
Khanderwal 2012	India		inpatient	Fried	≥60	unpecified	51	66.4 (6.3)	NA
Kitamura 2019	Japan		community	Fried	≥65	unspecified	176	71 (5.6)	730 (57.2%)
Lahousse 2014	Netherlands	Rotterdam Study	community	Fried	≥55	unspecified	211	74 (9)	NA
Lee 2017	Japan	National Center for Geriatrics and	community	Fried	≥65	unspecified	1218	73.6 (5.5)	5037 (52.4%)

		Gerontology – Study of Geriatric Syndromes.							
Lekan 2018	USA		inpatient	Frailty risk score	≥55	unspecified	136	70.1 (55-98)	146 (52.5%)
Li 2018	Taiwan	NHIS Taiwan	community	FRAIL	≥65	unspecified	719	NA	NA
Li 2019b	China	RuLAS	community	Fried	70-84	unspecified	121	73.3 (3.9)	937 (53.3%)
Li 2015	China		inpatient	FRAIL	≥60	Type 2	146	80 (74-84)	32 (21.9%)
Liccini 2016	USA		outpatient	FRAIL	≥50	unspecified	198	64.9 (8.7)	NA
Lin 2015	Taiwan	Taichung Community Health Study for Elders	community	Fried	≥65	unspecified	177	74 (7)	497 (48%)
MacKenzie 2019	Canada		inpatient	CFS	≥65	unspecified	141	81.4 (8.1)	228 (57%)
Matsuzawa 2010	Japan		inpatient	CGA	≥65	unspecified	288	72.8 (7.7)	164 (56.9%)
McAllister 2018	UK	United Kingdom Health Improvement Network Database	community	eFI	≥20	unspecified	292170	61.7	NA
McAllister 2016	USA	Clinformatics Data Mart	community	John Hopkins ACG	≥20	unspecified	191590	50.4 (9.9)	89151 (46.5%)
McAllister 2017	USA	Clinformatics Data Mart	community	John Hopkins ACG	≥20	unspecified	99694	53.9 (9.7)	NA
McClure 2019	Australia		community	SPPB	≥50	Type 2	87	70.2 (8.2)	29 (33.3%)
Merchant 2017	Singapore	HOPE study	community	FRAIL	≥65	unspecified	250	71.2	601 (57.2%)
Mohr 2007	USA	MMAS	community	Fried	≥50	unspecified	65	67.9 (6)	0 (0%)
Molist-Brunet 2019	Spain		inpatient	FI	≥85	Type 2	210	86.1 (4.8)	116 (55.2%)

Moreira 2017	Brazil		community	Fried	≥65	unspecified	855	74 (6)	2951 (66.3%)
Moreira 2016	Brazil	FIBRA study	community	Fried	≥65	Type 2	99	72	99 (100%)
Motokawa 2018	Japan		community	Kihon	≥65	unspecified	68	73.3 (5.8)	397 (59.7%)
Nadruz 2017	USA	ARIC	community	Fried	≥68	unspecified	1188	75.6 (5)	2355 (59%)
Nelson 2007	USA		outpatient	VES-13	≥75	unspecified	111	78	56 (50.5%)
Ng 2014	Singapore	Singapore Longitudinal Aging Study	community	Fried	≥55	unspecified	349	66.7 (7.7)	1084 (64.3%)
Nguyen 2019	Vietnam		community	Fried	≥60	unspecified	24	72.8 (8.2)	358 (68.5%)
Nguyen 2019b	New Zealand		outpatient	Fried	≥60	unspecified	158	69.5 (6.8)	98 (62%)
Nishimura 2019	Japan		outpatient	Kihon	≥60	Type 2	213	70.2 (5.5)	105 (49.3%)
Orkaby 2019	USA	Framingham Heart study	community	Fried	≥60	unspecified	350	69.7 (7)	1194 (55%)
Ottenbacher 2009	USA	H-EPESE study	community	Fried	≥65	unspecified	568	74.3 (6.4)	1195 (58.3%)
Pollack 2017	USA		community	Fried	≥65	unspecified	529	73.4 (5.8)	0 (0%)
Ricci 2014	Brazil	FIBRA study	community	Fried	≥65	unspecified	189	71.9 (5.9)	489 (64.3%)
Simpson 2016	USA		community	John Hopkins ACG	≥20	Type 2	54505	60 (52-68)	26380 (48.4%)
Sirola 2011	Finland	Helsinki Businessmen Study	community	Fried	≥65	unspecified	89	73 (73)	NA
Tamura 2018	Japan		outpatient	Fried		unspecified	185	78 (75-82)	201 (62.2%)
Tang 2013	China	BLSA-II	community	FI	≥55	unspecified	456	70.1 (9)	NA
Tepper 2018	Israel		outpatient	Fried	≥60	Type 2	117	70.6 (6.5)	46 (39.3%)

Thein 2018	Singapore	Singapore Longitudinal Ageing Study	community	Fried	≥55	Type 2	486	66 (7.6)	1693 (62.8%)
Tuttle 2018	USA		outpatient	mPPT	NA	Type 2	95	57 (12)	34 (35.8%)
Vaingankar 2017	Singapore	Well-being of the Singapore Elderly study	community	Fried	≥60	unspecified	622	69	1134 (53.9%)
van Hateren 2015	Netherlands	ZODIAC	outpatient	RAND-36	≥75	Type 2	858	72.3 (7.2)	519 (60.5%)
Vaz Fragozo 2009	USA		community	Fried	≥78	unspecified	75	84.3 (4.5)	252 (67.4%)
Veronese 2017	Iceland	Age, Gene/Environment Susceptibility (AGES)—Reykjavik Study	community	Fried	≥65	unspecified	427	76.2 (5.6)	2444 (64%)
Wang 2017	USA	Veterans Administration Electronic Medical Records	community	Frailty risk class	≥65	Type 2	41204	74.6 (5.8)	0 (0%)
Watanabe 2017	Japan	Obu Study of Health Promotion for the Elderly	community	Fried	≥60	unspecified	623	72.1 (5.6)	2446 (51.8%)
Weinstein 2018	Israel		community	Fried	45-74	unspecified	118	77.2 (6.4)	0 (0%)
Wong 2010	Canada	Montreal Unmet Needs Study	community	Fried	≥75	unspecified	125	79.6 (4)	502 (67.8%)
Woo 2019	China		community	FRAIL		unspecified	86	74.7 (7.7)	NA
Wu 2009	United States		inpatient	Fried	≥60	unspecified	14	77 (6)	NA

Wu 2018	China	Chinese Health and Retirement Longitudinal Study	community	Fried	≥65	unspecified	382	67	2618 (49.4%)
Xue 2019	China		inpatient	Fried	≥60	unspecified	36	78.5 (9)	NA
Yanagita 2018	Japan		outpatient	CFS	≥65	Type 2	132	78.3 (7.9)	NA

Outcomes of included studies

Mortality

Author	Year	Country	Setting	Frailty measure	Number with diabetes	Mean or median age (sd or IQR)	Analysis	Adjustment	Effect size
Cacciatore	2013	Italy	community	Frailty staging system	188	72.8 (5.8)	Cox model per tertile increase in frailty staging system (female)	age, BMI, waist circumference, heart rate, pulse blood pressure, Charlson comorbidity index, drugs number, GDS, insulin, hypoglycemic drugs, hypertension, CAD, CHF, PAD, and CKD.	HR 1.31 (1.03-1.85)
Cacciatore	2013	Italy	community	Frailty staging system	188	72.8 (5.8)	Cox model per unit increase in frailty staging system (males)	age, BMI, waist circumference, heart rate, pulse blood pressure, Charlson comorbidity index,	HR 1.99 (1.75-3.05)

								drugs number, GDS, insulin, hypoglycemic<br><br>drugs, hypertension, CAD, CHF, PAD, and CKD.	
Castro-Rodriguez	2016	Spain	community	FI	363	76 (71.2-79)	Cox model per 0.1 increase in FI	age, sex, Charlson index, disability	HR 1.83 (1.49-2.26)
Castro-Rodriguez	2016	Spain	community	Frailty trait scale	363	76 (71.2-79)	Cox model per 10% increase in scale	age, sex, Charlson index, disability	HR 1.51 (1.29-1.78)
Chao	2018	Taiwan	community	FRAIL	560795	56.4 (13.8)	Cox model (categorical on 0, 1, 2, 3+ FRAIL scale indicators)	Adjusted for demographic profiles, comorbidities (including obesity, mental illnesses, hypoglycemia history), substance use (smoking and alcohol abuse), aDCSI, and medications	HR 1.25 (1.15-1.36)
Chode	2016	USA	community	FRAIL	222	57.43 (4.4)	Logistic regression 9 years follow-up	NA	1.45 (1.12-1.86)

Ferri-Guerra	2019	USA	community	FI	763	72.87 (6.78)	Cox model, frail (FI>0.21) versus non-frail (FI<0.21)	adjusted for age, race, ethnicity, BMI and Median Household Income, Charlson Comorbidity Index, diabetes complications, duration of diabetes, use of insulin or sulfonylureas, metformin and level of glycemia control.	HR 2.65 (1.52-4.64)
Hubbard	2010	England	community	CFS	310	81.3	Cox regression	Age, sex, place of residence	1.42 (1.2-1.69)
Kitamura	2019	Japan	community	Fried	176	NA	Cox model (categorical)	age, sex, hypertension, high total cholesterol, low total cholesterol, low estimated glomerular filtration rate, overweight, low body mass index, anemia, hypoalbuminemia, low Mini-Mental State Examination score, history of	6.6 (2-22)

								stroke and current smoking	
Li	2015	NA	NA	FRAIL	NA	NA	Log rank test	none	Significant assocaition with frailty
Liccini	2016	USA	outpatient	FRAIL	198	64.9 (8.7)	Raw numbers of deaths only	NA	NA
Presley	2019	USA	inpatient	FI	500	65 (58-75)	Cox model (continuous, with example of 0.05 point increase)	demographics, administrative, clinical EHR data	1.45 (1.32-1.6)
Thein	2018	Singapore	community	Fried	486	67.3 (7.5)	Cox (frail versus not frail)	age, gender, education level, smoking, alcohol intake, and physical exercise, diabetes duration, WC, total cholesterol, HDL cholesterol, hypertension, cardiac disease, stroke, arthritis, hip fracture, polypharmacy,<br><br>and depression.	4.37 (2.38-8.03)
Wang	2017	USA	community	indicator diagnoses	41204	74.6 (5.8)	Cox regression	age, race/ethnicity, Charlson	0.98 (0.89-1.07)

								comorbidity score, BMI, HbA1c, statin use	

Hospitalisation and Emergency Department Attendance

Author	Year	Country	Setting	Frailty measure	Number with diabetes	Mean or median age (SD or IQR)	Outcome	Analysis	Covariate adjustment	Effect of frailty
Ferri-Guerra	2019	USA	community	FI	763	72.87 (6.78)	hospitalisation	CoxPH - prospective	adjusted for age, race, ethnicity, BMI and Median Household Income, Charlson Comorbidity Index, diabetes complications, duration of diabetes, use of insulin or sulfonylureas, metformin and level of glycemia control.	HR 2.36 (1.77-3.14)
Li	2018	Taiwan	community	FRAIL	719	NA	hospitalisation	Retrospective (event in past year)	Adjusted for age, sex, education, marital status, duration of diabetes, use of insulin, falls, ADl disability, and IADl disability	OR 5.31 (1.87-15.1)
Li	2015	China	inpatient	FRAIL	146	80 (74-84)	hospitalisation	prospective - logistic regression (3 or more hospitalisations in 1 year follow-up)	Adjusted for age, gender, MMSE points, BMI, duration of diabetes, HbA1c, macroangiopathy, and nephropathy	OR 5.99 (1.38-25.91)

Liccini	2016	USA	outpatient	FRAIL	198	64.9 (8.7)	hospitalisation	prospective - logistic regression (hospitalisation at 6 month follow-up)	adjusted for age, sex, education and HbA1c	OR 4.7 (1.67-13.19)
Chao	2018	Taiwan	community	FRAIL	560795	56.4 (13.8)	hospitalisation	CoxPH - prospective	Adjusted for demographic profiles, comorbidities (including obesity, mental illnesses, hypoglycemia history), substance use (smoking and alcohol abuse), aDCSI, and medications	HR 1.25 (1.17-1.33)
Li	2018	Taiwan	community	FRAIL	719	NA	ED visit	Retrospective (event in past year)	Adjusted for age, sex, education, marital status, duration of diabetes, use of insulin, falls, ADl disability, and IADl disability	4.05 (1.31-12.49)

HbA1c

Author	Year	Country	Setting	Frailty measure	Number with diabetes	Mean or median age (SD or IQR)	Outcome	Analysis	Result
Atif	2019	Pakistan	outpatient	CFS	400	NA	HbA1c above target level (7%) or below)	adjusted logistic regression	No significant difference with frailty (1.11 (0.44, 2.84)
Ferri-Guerra	2019	USA	community	FI	763	72.87 (6.78)	HbA1c categorised as tight (<7%) intermediate (7-9%) and poor (>9%)	Chi squared	No significant difference with frailty
van Hateren	2015	Netherlands	outpatient	RAND-36	858	72.3 (7.2)	mean HbA1c	mean difference	No significant difference between frail and non-frail
Kitamura	2019	Japan	community	Fried	176	NA	mean HbA1c	mean difference	No significant difference between frail and non-frail
Li	2015	China	inpatient	Fried	146	80 (74-84)	mean HbA1c	mean difference	No significant

									difference between frail and non-frail
MacKenzie	2019	Canada	inpatient	CFS	141	80.6 (7.8)	mean HbA1c	mean difference	No significant difference between frail and non-frail
Matsuzawa	2010	Japan	inpatient	CGA	288	72.8 (7.7)	mean HbA1c	mean difference (t-test)	Higher HbA1c in frail group (7.9±1.1 vs 7.4±1.4)
McAlister	2016	NA	NA	John Hopkins ACG	NA	NA	HbA1c categorised as <7%, 7-8%, 8-9% and >9%	descriptive	no difference between health status groups
McAlister	2017	NA	NA	John Hopkins ACG	NA	NA	HbA1c categorised as <7%, 7-8%, 8-9% and >9%	Chi squared	Slightly higher proportion of frail in <7% group and in >9 group
McAlister	2018	NA	NA	eFI	NA	NA	HbA1c categorised as <6%, 6-6.5%, 6.5-	Chi squared	higher proportion of frail in <6% group, lower

							7%, 7-7.5% and >7.5%		proportion of frail in >7.5 group
Molist-Brunet	2019	Spain	inpatient	FI	210	86.1 (4.8)	mean HbA1c	mean difference (not satistically tested by no clinically meaningful difference between frailty groups)	not satistically tested by no clinically meaningful difference between frailty groups
Nelson	2007	USA	outpatient	VES-13	111	78	mean HbA1c	mean difference	no significant difference
Nelson	2007	USA	outpatient	VES-13	111	78	HbA1c <7%	Chi squared	no significant difference
Yanagita	2018	Japan	outpatient	CFS	132	78.3 (7.9)	mean HbA1c	mean difference	lower with frailty - lowest HbA1c at most severe end of frailty spectrum

Diabetes complications

Author	Year	Country	Setting	Frailty_measure	N_diabetes	age	Outcome	Description	
Pilotto	2014	Italy	Outpatient	CGA	1324	73.3 (5.6)	cerebrovascular disease	Chi squared test for cross sectional assocation between frailty and cerebrovascular disease	Positive association
Pilotto	2014	Italy	Outpatient	CGA	1324	73.3 (5.6)	coronary artery disease	Chi squared test for cross sectional assocation between frailty and coronary artery disease	Positive association
Pilotto	2014	Italy	Outpatient	CGA	1324	73.3 (5.6)	any event in last 3 months	Logistic regression (OR 1.83 (1.17, 2.86)	Positive association
Pilotto	2014	Italy	Outpatient	CGA	1324	73.3 (5.6)	Hypoglycaemic hospitalisation	Logistic regression (OR 7.67 (3.32, 17.7)	Positive association
Simpson	2016	USA	community	John Hopkins ACG	54505	60 (52-68)	New macrovascular complication	Multivariate Cox Regression Analysis of New Diabetes	no association

								Complication in 54 505 Patients Initiating Oral Antidiabetic Drugs. HR 0.99 (0.86-1.13)	
van Hateren	2015	Netherlands	outpatient	RAND-36	858	72.3 (7.2)	Macrovascular complications	Chi squared test for cross sectional assocation between frailty and macrovascular disease	Positive association
Chao	2018	Taiwan	community	FRAIL	560795	56.4 (13.8)	cardiovascular event	Cox PH model: HR 1.13 (1.02-1.25)	Positive association
Li	2015	China	inpatient	FRAIL	146	80 (74-84)	macroangiopathy	Logistic regression (OR 0.87 (0.24-3.13)	no association
Hubbard	2010	England	community	CFS	310	81.3	Complications (retinopathy, recurrent infections, nephropathy and peripheral neuropathy)	Among older adults with diabetes, those who were frail were<br><br>2.62 times more likely to have a complication of diabetes than those who	Positive association

								were not frail (95% CI 1.36–5.06 times). This was independent of age, sex andnumber of years livingwith diabetes.	
Simpson	2016	USA	community	John Hopkins ACG	54505	50 (62-58)	New microvascular complication	Multivariate Cox Regression Analysis of New Diabetes Complication in 54 505 Patients Initiating Oral Antidiabetic Drugs. HR 0.89 (0.70-1.13)	no associatio n
Ferri-Guerra	2019	USA	community	FI	763	72.87 (6.78)	Diabetes with End organ damage: patients diagnosed with one or more of the following diagnosis: retinopathy, neuropathy and nephropathy.	Chi squared test for cross sectional assocation between frailty and microvascular disease	Positive associatio n
Chao	2018	Taiwan	community	FRAIL	560795	56.4 (13.8)	aDCSI scores	Chi squared test for cross sectional	Positive associatio n

								assocation between frailty and aDCSI scores	
McAllister	2018	UK	community	eFI	292170	61.7	nephropathy	Chi squared test for cross sectional assocation between frailty and nephropathy	Positive association
Pilotto	2014	Italy	Outpatient	CGA	1324	73.3 (5.6)	nephropathy	Chi squared test for cross sectional assocation between frailty and nephropathy	Positive association
Adame Perez	2019	Canada	outpatient	Edmonton	41	70 (65-74)	nephropathy	Chi squared test for cross sectional assocation between frailty and nephropathy	Positive association
Li	2015	China	inpatient	FRAIL	146	80 (74-84)	nephropathy	Logistic regression (OR 4.46 (1.24-15.97)	Positive association
Tuttle	2018	USA	outpatient	mPPT	95	57 (12)	Peripheral neuropathy	Chi squared test for cross sectional assocation	Positive association

								between frailty and peripheral neuropathy	
McAllister	2018	UK	community	eFI	292170	61.7	neuropathy	Chi squared test for cross sectional assocation between frailty and neuropathy	Positive association
Nelson	2007	USA	outpatient	VES-13	111	78	neuropathy	Chi squared test for cross sectional assocation between frailty and neuropathy	Positive association
Pilotto	2014	Italy	Outpatient	CGA	1324	73.3 (5.6)	neuropathy	Chi squared test for cross sectional assocation between frailty and neuropathy	Positive association
McAllister	2018	UK	community	eFI	292170	61.7	retinopathy	Chi squared test for cross sectional assocation between frailty and retinopathy	Positive association

Nelson	2007	USA	outpatient	VES-13	111	78	visual impairment	Chi squared test for cross sectional assocation between frailty and retinopathy	Positive associatio n
Pilotto	2014	Italy	Outpatient	CGA	1324	73.3 (5.6)	retinopathy	Chi squared test for cross sectional assocation between frailty and retinopathy	Positive associatio n
Boas	2018	Brazil	outpatient	Edmonton	100	NA	foot ulcer	Increasing frailty severity assocaited with higher proportion of participants with foot ulceration	Positive associatio n

Disability

**Author**	**Yea r**	**Country**	**Setting**	**Frailty measure**	**Numb er with diabet es**	**Mean or media n age (SD or IQR)**	**Disability measure**	**Covariate adjustment**	**Analysis**	**Outcome**

Li	2015	China	inpatient	FRAIL	146	80 (74-84)	Physical performance was assessed by activitied of daily living (ADLs) and instrumental activities of fraily living (IADLs). ADLs included 6 items: feeding, bowels and bladder control, toileting, transfers, dressing and bathing. IADLs included 8 items: using the telephone, food perparation, shopping, managing money, house-keeping, laundry, getting to places outside of walking distance, and taking medicine. Each of the items was caegorized as severely dependent, assistant living, and completely independent. ADL disability and IADL disability were defined as requiring any assistance in performing at least 1 of the items, respectively.	models adjusted for age, gender, MMSE points, BMI, duration of diabetes and HbA1c	logistic regression	cross sectional association (OR 6.58 (1.66-26.10) for ADL)

Chode	2016	USA	community	FRAIL	222	57.43 (4.4)	ADLs included seven items (bathing, dressing, eating, transferring bed or chair, walking across a room, getting outside, and using toilet) (21). IADLs included eight items (preparing meals, shopping for groceries, managing money, making phone calls, doing light housework, doing heavy housework, getting to places outside walking distance, and managing medications)	age, sex	linear regression (number of ADL/IADL impairments)	cross sectional associations (number of impairments)

Castro-Rodriguez	2016	Spain	community	FI	363	76 (71.2-79)	Functional disability was evaluated according to the Katz Index<br><br>(which ranks dependency 6six functions of daily living: bathing, dressing, toileting, transferences, continence, and feeding) obtained from each individual in the baseline and follow-up visits.24 Incident disability was ascertained by comparison of the Katz Index. People were classified as having incident disability when any worsening in the Katz Index was detected	none	Baseline FI compared between those with and without incident disability	Positive association between frailty and incident disability

Liccini	2016	USA	outpatient	FRAIL	198	64.9 (8.7)	ADLs<br><br>included seven items (bathing, dressing, eating, transferring<br><br>bed or chair, walking across a room, getting outside, and using<br><br>toilet) (21). IADLs included eight items (preparing meals,<br><br>shopping for groceries, managing money, making phone calls,<br><br>doing light housework, doing heavy housework, getting to<br><br>places outside walking distance, and managing medications)	age, sex, education and HbA1c	logistic regression	new disability (OR for frailty 3.57 (1.27-10.04)

Thein	2018	Singapore	community	Fried	486	67.3 (7.5)	Dependency on daily living activities was measured using self reported difficulty or needing assistance in instrumental activities of daily living (IADL) and activities of daily living (ADL), as previously validated in a local cohort	Adjusted for age, gender, education level, smoking, alcohol intake, and physical exercise, diabetes duration, WC, total cholesterol, HDL cholesterol, hypertension, cardiac disease, stroke, arthritis, hip fracture, polypharmacy, and depression.	logistic regression	cross sectional associations with ADL/IADL disability (OR 20.2 (7.74–52.6)

Cognitive impairment

Author	Year	Country	Setting	Frailty measure	Number with diabetes	Mean or median age (SD or IQR)	Measure	Cross sectional/ prospective	Analysis	Result
Bello-Chavolla	2017	Mexico	community	Fried	135	77.7 (5.8)	MMSE	cross sectional	Chi squared	positive association
Matsuzawa	2010	Japan	inpatient	CGA	288	72.8 (7.7)	MMSE	cross sectional	Chi squared	positive association
Cacciatore	2013	Italy	community	Frailty staging system	188	72.8 (5.8)	MMSE	cross sectional	multivariable logistic regression model for frailty	positive association

Quality of Life

Author	Year	Country	Setting	Frailty measure	Number with diabetes	Mean or median age (SD or IQR)	Cross sectional/ prospective	Analysis	Result
Adame Perez	2019	Canada	outpatient	Edmonton	41	70 (65-74)	cross sectional	Participants with frailty scored a median (range) of 31 (13 to 54) points lower in HRQoL	positive association

								scores when compared to nonfrail participants (p?0.05)	
Matsuzawa	2010	Japan	inpatient	CGA	288	72.8 (7.7)	cross sectional	Chi squared test comparing frail and non frailty people and QOL scores	positive association
Nguyen	2019	Vietnam	community	Fried	24	NA	cross sectional	The mean EQ-5D-5L indexes of the non-frailty, pre-frailty, and frailty groups were 0.70 (SD = 0.18), 0.70 (SD = 0.19), and 0.58 (SD = 0.20), respectively. The differences were found between non-frailty and frailty groups (p <	positive association

								0.01), as well as the pre-frailty and frailty groups (p<0.01).	

Depression

**Author**	**Year**	**Country**	**Setting**	**Frailty measure**	**Number with diabetes**	**Mean or median age (SD or IQR)**	**Cross sectional/ prospective**	**Analysis**	**Result**	
Almeida	2016	Australia	community	FRAIL	717	NA	Geriatric depression scale	cross sectional	OR for current depression 8.92 (7.10, 11.20)	positive association
Adame Perez	2019	Canada	outpatient	Edmonton	41	70 (65-74)	Major depression inventory	cross sectional	Frail participants had a higher incidence (83% frail vs. 6% non frail) of depression (p=0.005) than those without frailty	positive association

Matsuzawa	2010	Japan	inpatient	CGA	288	72.8 (7.7)	Geriatric depression scale	cross sectional	No significant difference in mean score	no association

Assessment of publication bias

Funnel plot of studies assessing relationship between diabetes status and incidence of frailty

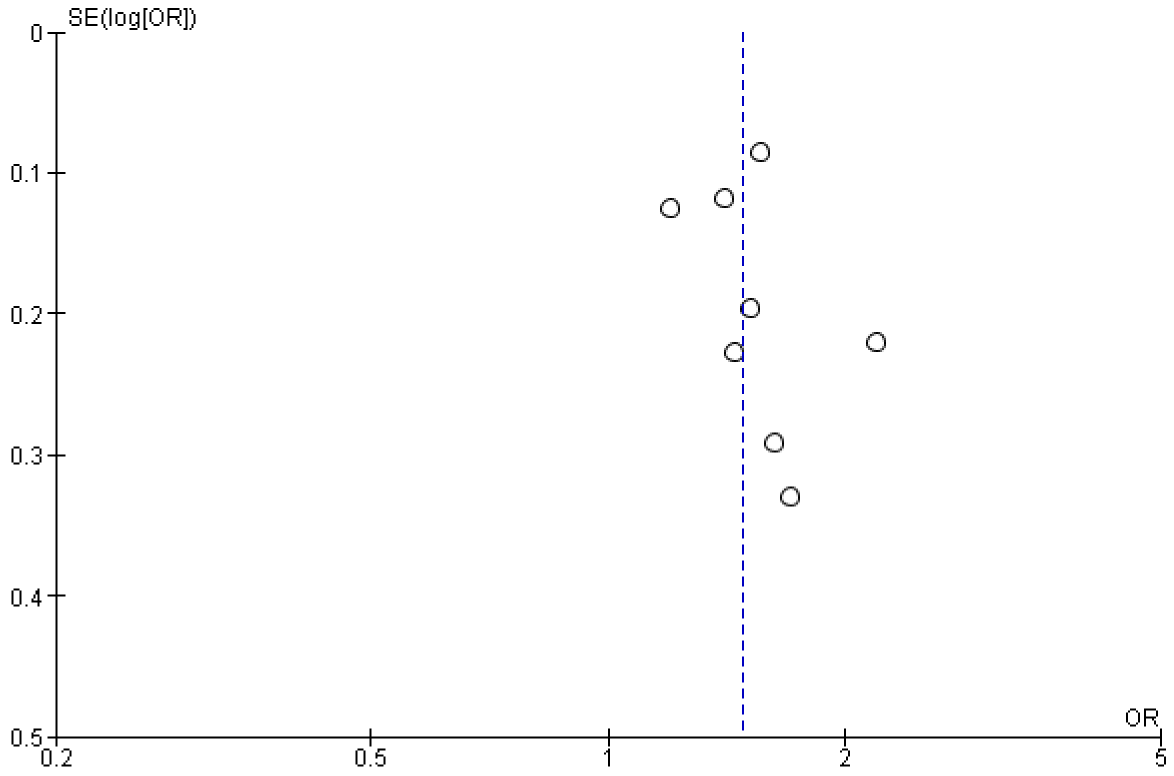

No clear evidence of publication bias. However, this should be interpreted with caution given the small number of studies included in the plot.

Funnel plot of studies assessing hazard ratio of mortality associated with frailty

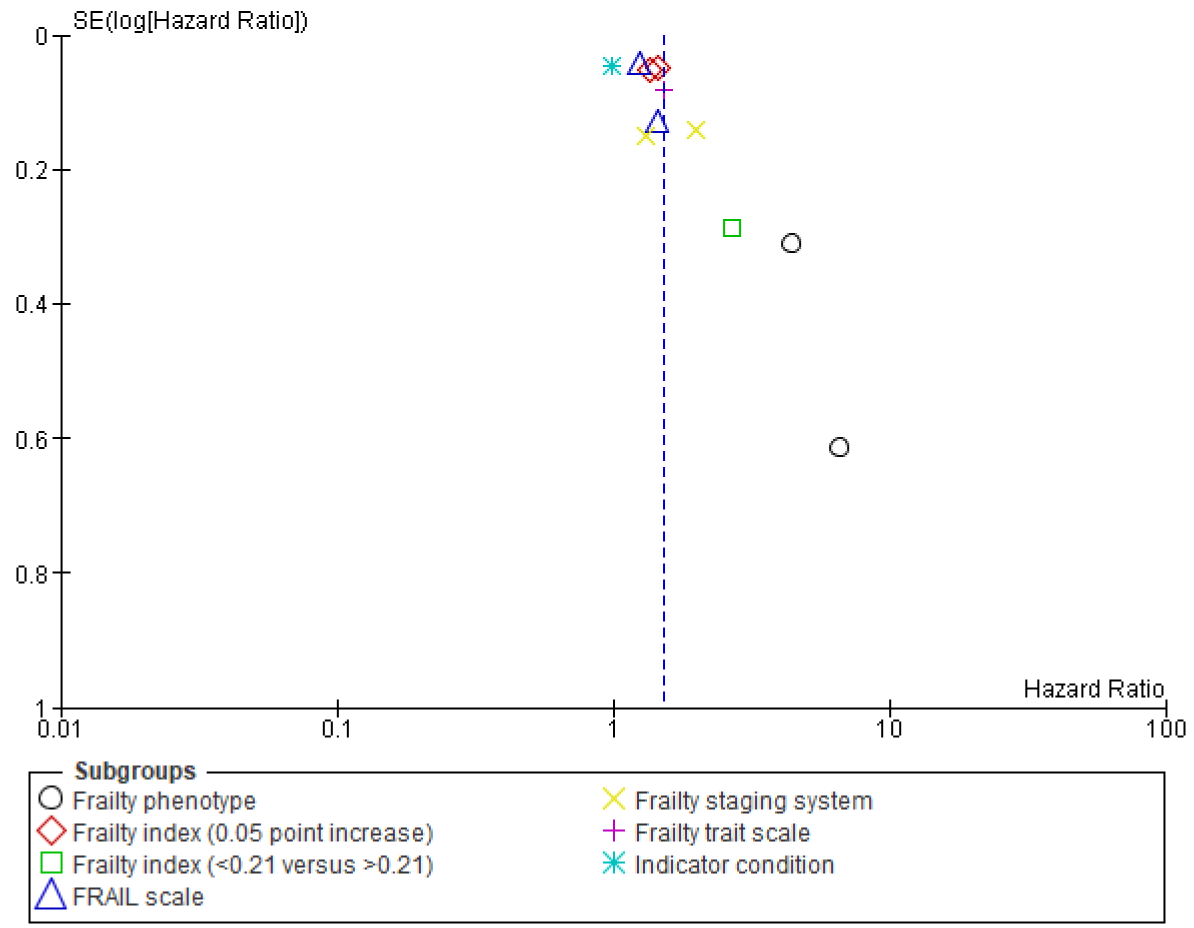

Some asymmetry in this plot which may include publication bias. However this appears to be driven by the higher effect size of the frailty phenotype studies rather than true bias towards studies with higher effect sizes.

References of included studies

Studies included in figure 2 are indicated in bold.

1. Adame Perez SI, Senior PA, Field CJ, Jindal K, Mager DR. Frailty, Health-Related Quality of Life, Cognition, Depression, Vitamin D and Health-Care Utilization in an Ambulatory Adult Population With Type 1 or Type 2 Diabetes Mellitus and Chronic Kidney Disease: A Cross-Sectional Analysis. *Canadian journal of diabetes* 2019; 43(2): 90-7.

2. Aguayo GA, Hulman A, Vaillant MT, et al. Prospective association among diabetes diagnosis, HbA1c, glycemia, and frailty trajectories in an elderly population. *Diabetes Care* 2019; 42(10): 1903-11.

3. Aguilar-Navarro SG, Mimenza-Alvarado AJ, Corona-Sevilla I, et al. Cerebral vascular reactivity in frail older adults with vascular cognitive impairment. *Brain Sciences* 2019; 9(9): 214.

4. Al Snih S, Graham JE, Ray LA, Samper-Ternent R, Markides KS, Ottenbacher KJ. Frailty and incidence of activities of daily living disability among older Mexican Americans. *Journal of Rehabilitation medicine* 2009; 41(11): 892-7.

5. Almeida OP, McCaul K, Hankey GJ, et al. Duration of diabetes and its association with depression in later life: The Health In Men Study (HIMS). *Maturitas* 2016; **86**: 3-9.

6. Ambagtsheer RC, Beilby J, Seiboth C, Dent E. Prevalence and associations of frailty in residents of Australian aged care facilities: findings from a retrospective cohort study. *Aging Clin Exp Res* 2019.

7. Anjos DMD, Moreira BD, Pereira DS, et al. Impact of Type-2 Diabetes Time Since Diagnosis on Elderly Women Gait and Functional Status. *Physiother Res Int* 2017; 22(2): 8.

8. Atif M, Saleem Q, Asghar S, Malik H, Ahmad N. Extent and Predictors of Poor Glycaemic Control among Elderly Pakistani Patients with Type 2 Diabetes Mellitus: A Multi-Centre Cross-Sectional Study. *Med Lith* 2019; 55(1): 11.

9. Avila-Funes JA, Helmer C, Amieva H, et al. Frailty among community-dwelling elderly people in France: the three-city study. *The Journals of Gerontology Series A: Biological Sciences and Medical Sciences* 2008; 63(10): 1089-96.

10. Azmon M, Gayus N, Michal H, Olmer L, Cukierman-Yaffe T. The Association between Glucose Control and Functional Indices in Older People with Diabetes. *Int J Endocrinol* 2018: 7.

11. Bello-Chavolla OY, Aguilar-Salinas CA, Avila-Funes JA. Geriatric Syndromes and Not Cardiovascular Risk Factors are Associated with Cognitive Impairment among Mexican Community-Dwelling Elderly with Type 2 Diabetes. *Revista de investigacion clinica; organo del Hospital de Enfermedades de la Nutricion* 2017; 69(3): 166-72.

12. Boas N, Salome GM, Ferreira LM. Frailty syndrome and functional disability among older adults with and without diabetes and foot ulcers. *J Wound Care* 2018; 27(7): 409-16.

13. Bouillon K, Kivimaki M, Hamer M, et al. Diabetes risk factors, diabetes risk algorithms, and the prediction of future frailty: the Whitehall II prospective cohort study. *Journal of the American Medical Directors Association* 2013; **14**(11): 851.e1-6.

14. Brunner EJ, Shipley MJ, Ahmadi-Abhari S, et al. Midlife contributors to socioeconomic differences in frailty during later life: a prospective cohort study. *The Lancet Public Health* 2018; **3**(7): e313-e22.

15. Cacciatore F, Testa G, Galizia G, et al. Clinical frailty and long-term mortality in elderly subjects with diabetes. *Acta diabetologica* 2013; 50(2): 251-60.

16. Cakmur H. Frailty among elderly adults in a rural area of Turkey. *Medical science monitor : international medical journal of experimental and clinical research* 2015; 21: 1232-42.

17. Calado LB, Ferriolli E, Moriguti JC, Martinez EZ, Lima NKD. Frailty syndrome in an independent urban population in Brazil (FIBRA study): a cross-sectional populational study. *Sao Paulo Med J* 2016; 134(5): 385-92.
18. Carneiro JA, Ramos GCF, Barbosa ATF, de Mendonca JMG, da Costa FM, Caldeira AP. Prevalence and factors associated with frailty in non-institutionalized older adults. *Rev Bras Enferm* 2016; 69(3): 408-15.
19. Castrejon-Perez RC, Aguilar-Salinas CA, Gutierrez-Robledo LM, Cesari M, Perez-Zepeda MU. Frailty, diabetes, and the convergence of chronic disease in an age-related condition: a population-based nationwide cross-sectional analysis of the Mexican nutrition and health survey. *Aging Clin Exp Res* 2018; 30(8): 935-41.
20. Castrejón-Pérez RC, Borges-Yáñez SA, Gutiérrez-Robledo LM, Ávila-Funes JA. Oral health conditions and frailty in Mexican community-dwelling elderly: a cross sectional analysis. *BMC public health* 2012; 12(1): 773.
21. Castrejon-Perez RC, Gutierrez-Robledo LM, Cesari M, Perez-Zepeda MU. Diabetes mellitus, hypertension and frailty: A population-based, cross-sectional study of Mexican older adults. *Geriatr Gerontol Int* 2017; **17**(6): 925-30.
22. Castro-Rodriguez M, Carnicero JA, Garcia-Garcia FJ, et al. Frailty as a Major Factor in the Increased Risk of Death and Disability in Older People With Diabetes. *Journal of the American Medical Directors Association* 2016; **17**(10): 949-55.
23. Cesari M, Leeuwenburgh C, Lauretani F, et al. Frailty syndrome and skeletal muscle: results from the Invecchiare in Chianti study. *The American journal of clinical nutrition* 2006; 83(5): 1142-8.
24. Chang SS, Weiss CO, Xue Q-L, Fried LP. Patterns of comorbid inflammatory diseases in frail older women: the Women's Health and Aging Studies I and II. *Journals of Gerontology Series A: Biomedical Sciences and Medical Sciences* 2010; 65(4): 407-13.
25. Chang Y-W, Chen W-L, Lin F-G, et al. Frailty and its impact on health-related quality of life: a cross-sectional study on elder community-dwelling preventive health service users. *PloS one* 2012; 7(5).
26. Chao CT, Wang J, Chien KL, Ntuh COGN. Both pre-frailty and frailty increase healthcare utilization and adverse health outcomes in patients with type 2 diabetes mellitus. *Cardiovasc Diabetol* 2018; 17: 13.
27. Chaves PHM, Semba RD, Leng SX, et al. Impact of anemia and cardiovascular disease on frailty status of community-dwelling older women: the Women's Health and Aging Studies I and II. *The Journals of Gerontology Series A: Biological Sciences and Medical Sciences* 2005; 60(6): 729-35.
28. Chen C-Y, Wu S-C, Chen L-J, Lue B-H. The prevalence of subjective frailty and factors associated with frailty in Taiwan. *Archives of gerontology and geriatrics* 2010; 50: S43-S7.
29. Chen LJ, Chen CY, Lue BH, Tseng MY, Wu SC. Prevalence and associated factors of frailty among elderly people in Taiwan. *International Journal of Gerontology* 2014; 8(3): 114-9.
30. Cheong CY, Nyunt MSZ, Gao Q, et al. Risk Factors of Progression to Frailty: Findings from the Singapore Longitudinal Ageing Study. *Journal of Nutrition, Health and Aging* 2019.
31. Chhetri JK, Zheng Z, Xu X, Ma C, Chan P. The prevalence and incidence of frailty in Pre-diabetic and diabetic community-dwelling older population: results from Beijing longitudinal study of aging II (BLSA-II). *BMC geriatrics* 2017; 17(1): 47.
32. Chiu CJ, Cheng YY. Utility of geriatric syndrome indicators for predicting subsequent health care utilization in older adults in Taiwan. *International Journal of Environmental Research and Public Health* 2019; **16**(3): 456.
33. Chode S, Malmstrom TK, Miller DK, Morley JE. Frailty, Diabetes, and Mortality in Middle-Aged African Americans. *The journal of nutrition, health & aging* 2016; **20**(8): 854-9.
34. Cigolle CT, Ofstedal MB, Tian Z, Blaum CS. Comparing models of frailty: the Health and Retirement Study. *Journal of the American Geriatrics Society* 2009; 57(5): 830-9.

35. Crow RS, Lohman MC, Titus AJ, et al. Mortality Risk Along the Frailty Spectrum: Data from the National Health and Nutrition Examination Survey 1999 to 2004. *Journal of the American Geriatrics Society* 2018; 66(3): 496-502.
36. da Silva AP, da Pureza DY, Landre CB. Frailty syndrome in elderly patients with type 2 diabetes mellitus. *Acta Paul Enferm* 2015; 28(6): 503-9.
37. Danon-Hersch N, Rodondi N, Spagnoli J, Santos-Eggimann B. Prefrailty and chronic morbidity in the youngest old: an insight from the Lausanne cohort Lc65+. *Journal of the American Geriatrics Society* 2012; 60(9): 1687-94.
38. de León González ED, Hermosillo HG, Beltran JAM, et al. Validation of the FRAIL scale in Mexican elderly: results from the Mexican Health and Aging Study. *Aging Clin Exp Res* 2016; 28(5): 901-8.
39. Doi T, Makizako H, Tsutsumimoto K, et al. Transitional status and modifiable risk of frailty in Japanese older adults: A prospective cohort study. *Geriatr Gerontol Int* 2018; **18**(11): 1562-6.
40. Espinoza SE, Hazuda HP. Frailty prevalence and neighborhood residence in older Mexican Americans: the San Antonio longitudinal study of aging. *Journal of the American Geriatrics Society* 2015; **63**(1): 106-11.
41. Espinoza SE, Jung I, Hazuda H. Lower frailty incidence in older Mexican Americans than in older European Americans: the San Antonio Longitudinal Study of Aging. *Journal of the American Geriatrics Society* 2010; **58**(11): 2142-8.
42. Espinoza SE, Jung I, Hazuda H. Frailty transitions in the San Antonio Longitudinal Study of Aging. *Journal of the American Geriatrics Society* 2012; **60**(4): 652-60.
43. Ferri-Guerra J, Aparicio-Ugarriza R, Salguero D, et al. The Association of Frailty with Hospitalizations and Mortality Among Community Dwelling Older Adults with Diabetes. *J Frality Aging*: 7.
44. Fried LP, Tangen CM, Walston J, et al. Frailty in older adults: evidence for a phenotype. *The Journals of Gerontology Series A: Biological Sciences and Medical Sciences* 2001; 56(3): M146-M57.
45. Fugate Woods N, LaCroix AZ, Gray SL, et al. Frailty: emergence and consequences in women aged 65 and older in the Women's Health Initiative Observational Study. *Journal of the American Geriatrics Society* 2005; 53(8): 1321-30.
46. Garcia-Esquinas E, Graciani A, Guallar-Castillon P, Lopez-Garcia E, Rodriguez-Manas L, Rodriguez-Artalejo F. Diabetes and risk of frailty and its potential mechanisms: a prospective cohort study of older adults. *Journal of the American Medical Directors Association* 2015; **16**(9): 748-54.
47. Hanlon P, Nicholl BI, Jani BD, Lee D, McQueenie R, Mair FS. Frailty and pre-frailty in middle-aged and older adults and its association with multimorbidity and mortality: a prospective analysis of 493 737 UK Biobank participants. *The Lancet Public health* 2018; 3(7): e323-e32.
48. Hasan SS, Kow CS, Verma RK, Ahmed SI, Mittal P, Chong DWK. An evaluation of medication appropriateness and frailty among residents of aged care homes in Malaysia: A cross-sectional study. *Medicine* 2017; 96(35): e7929.
49. Hippisley-Cox J, Coupland C. Development and validation of QMortality risk prediction algorithm to estimate short term risk of death and assess frailty: cohort study. *BMJ (Clinical research ed)* 2017; 358: j4208.
50. Howrey BT, Al Snih S, Markides KS, Ottenbacher KJ. Frailty and diabetes among Mexican American older adults. *Annals of epidemiology* 2018; **28**(7): 421-6.e1.
51. Hubbard RE, Andrew MK, Fallah N, Rockwood K. Comparison of the prognostic importance of diagnosed diabetes, co-morbidity and frailty in older people. *Diabetic medicine : a journal of the British Diabetic Association* 2010; 27(5): 603-6.
52. Hyde Z, Smith K, Flicker L, et al. HbA1c Is Associated with Frailty in a Group of Aboriginal Australians. *The Journal of frailty & aging* 2019; **8**(1): 17-20.
53. Khan H, Kalogeropoulos AP, Georgiopoulou VV, et al. Frailty and risk for heart failure in older adults: the health, aging, and body composition study. *American heart journal* 2013; 166(5): 887-94.

54. Khandelwal D, Goel A, Kumar U, Gulati V, Narang R, Dey AB. Frailty is associated with longer hospital stay and increased mortality in hospitalized older patients. *The journal of nutrition, health & aging* 2012; 16(8): 732-5.
55. Kirkwood RN, Borem IL, Sampaio RF, et al. Frailty Status and Gait Parameters of Older Women With Type 2 Diabetes. *Canadian journal of diabetes* 2019; **43**(2): 121-7.
56. Kitamura A, Taniguchi Y, Seino S, et al. Combined effect of diabetes and frailty on mortality and incident disability in older Japanese adults. *Geriatrics and Gerontology International* 2019; 19(5): 423-8.
57. Lahousse L, Maes B, Ziere G, et al. Adverse outcomes of frailty in the elderly: the Rotterdam Study. *European journal of epidemiology* 2014; 29(6): 419-27.
58. Lee JSW, Auyeung TW, Leung J, Kwok T, Leung PC, Woo J. Physical frailty in older adults is associated with metabolic and atherosclerotic risk factors and cognitive impairment independent of muscle mass. *The journal of nutrition, health & aging* 2011; **15**(10): 857-62.
59. Lee JSW, Auyeung T-W, Leung J, Kwok T, Woo J. Transitions in frailty states among community-living older adults and their associated factors. *Journal of the American Medical Directors Association* 2014; 15(4): 281-6.
60. Lee S, Harada K, Bae S, et al. Relationship between chronic kidney disease with diabetes or hypertension and frailty in community-dwelling Japanese older adults. *Geriatrics and Gerontology International* 2017; 17(10): 1527-33.
61. Lekan DA, McCoy TP. Frailty risk in hospitalised older adults with and without diabetes mellitus. *Journal of clinical nursing* 2018; 27(19-20): 3510-21.
62. Li CL, Stanaway FF, Lin JD, Chang HY. Frailty and health care use among community-dwelling older adults with diabetes: A population-based study. *Clinical Interventions in Aging* 2018; 13: 2295-300.
63. Li G, Prior JC, Leslie WD, et al. Frailty and risk of fractures in patients with type 2 diabetes. *Diabetes Care* 2019; **42**(4): 507-13.
64. Li J, Nie S, Wang S, et al. Operationalization of Frailty Index in Diabetic Older Patients. *Journal of the American Medical Directors Association* 2016; **17**(3): 273-4.
65. Li Y, Zou Y, Wang S, et al. A Pilot Study of the FRAIL Scale on Predicting Outcomes in Chinese Elderly People With Type 2 Diabetes. *Journal of the American Medical Directors Association* 2015; 16(8): 714.e7-.e12.
66. Li YX, Jiang XY, Stone C, et al. A new physical-cognitive scale for assessment of frailty in Chinese Han elderly. *Neurological Research* 2019; 41(8): 728-33.
67. Liccini A, Malmstrom TK. Frailty and Sarcopenia as Predictors of Adverse Health Outcomes in Persons With Diabetes Mellitus. *Journal of the American Medical Directors Association* 2016; 17(9): 846-51.
68. Lin CH, Chou CY, Liu CS, Huang CY, Li TC, Lin CC. Association between frailty and subclinical peripheral vascular disease in a community-dwelling geriatric population: Taichung Community Health Study for Elders. *Geriatrics and Gerontology International* 2015; 15(3): 261-7.
69. MacKenzie HT, Tugwell B, Rockwood K, Theou O. Frailty and Diabetes in Older Hospitalized Adults: The Case for Routine Frailty Assessment. *Canadian Journal of Diabetes* 2019.
70. Matsuzawa T, Sakurai T, Kuranaga M, Endo H, Yokono K. Predictive factors for hospitalized and institutionalized care-giving of the aged patients with diabetes mellitus in Japan. *The Kobe journal of medical sciences* 2011; 56(4): E173-83.
71. McAlister FA, Lethebe BC, Lambe C, Williamson T, Lowerison M. Control of glycemia and blood pressure in British adults with diabetes mellitus and subsequent therapy choices: a comparison across health states. *Cardiovasc Diabetol* 2018; 17(1): 27.
72. McAlister FA, Youngson E, Eurich DT. Treated glycosylated hemoglobin levels in individuals with diabetes mellitus vary little by health status: A retrospective cohort study. *Medicine* 2016; 95(24): e3894.

73. McAlister FA, Youngson E, Eurich DT. Treatment Deintensification Is Uncommon in Adults With Type 2 Diabetes Mellitus: A Retrospective Cohort Study. *Circulation Cardiovascular quality and outcomes* 2017; 10(4).
74. McClure R, Villain A. Greater adherence to a Mediterranean Diet is associated with better gait speed in older adults with type 2 diabetes mellitus. *Clin Nutr ESPEN* 2019; 32: 33-9.
75. Merchant RA, Chen MZ, Tan LWL, Lim MY, Ho HK, van Dam RM. Singapore Healthy Older People Everyday (HOPE) Study: Prevalence of Frailty and Associated Factors in Older Adults. *Journal of the American Medical Directors Association* 2017; 18(8): 734.
76. Mohr BA, Bhasin S, Kupelian V, Araujo AB, O'Donnell AB, McKinlay JB. Testosterone, sex hormone–binding globulin, and frailty in older men. *Journal of the American Geriatrics Society* 2007; 55(4): 548-55.
77. Molist-Brunet N, Sevilla-Sanchez D, Puigoriol-Juvanteny E, et al. Optimizing drug therapy in frail patients with type 2 diabetes mellitus. *Aging Clin Exp Res* 2019.
78. Moreira Bd.e S, Dos Anjos DM, Pereira DS, et al. The geriatric depression scale and the timed up and go test predict fear of falling in community-dwelling elderly women with type 2 diabetes mellitus: a cross-sectional study. *BMC geriatrics* 2016; 16: 56.
79. Moreira BdS, Sampaio RF, Diz JBM, et al. Factors associated with fear of falling in community-dwelling older adults with and without diabetes mellitus: Findings from the Frailty in Brazilian Older People Study (FIBRA-BR). *Experimental gerontology* 2017; 89: 103-11.
80. Motokawa K, Watanabe Y, Edahiro A, et al. Frailty Severity and Dietary Variety in Japanese Older Persons: A Cross-Sectional Study. *The journal of nutrition, health & aging* 2018; 22(3): 451-6.
81. Nadruz W, Jr., Kitzman D, Windham BG, et al. Cardiovascular Dysfunction and Frailty Among Older Adults in the Community: The ARIC Study. *The journals of gerontology Series A, Biological sciences and medical sciences* 2017; 72(7): 958-64.
82. Nelson JM, Dufraux K, Cook PF. The relationship between glycemic control and falls in older adults. *Journal of the American Geriatrics Society* 2007; 55(12): 2041-4.
83. Ng TP, Feng L, Nyunt MSZ, Larbi A, Yap KB. Frailty in older persons: multisystem risk factors and the Frailty Risk Index (FRI). *Journal of the American Medical Directors Association* 2014; 15(9): 635-42.
84. Nguyen AT, Nguyen LH, Nguyen TX, et al. Frailty prevalence and association with health-related quality of life impairment among rural community-dwelling older adults in Vietnam. *International Journal of Environmental Research and Public Health* 2019; 16(20): 3869.
85. Nguyen TTH, Vu HTT, Nguyen TN, et al. Assessment of nutritional status in older diabetic outpatients and related factors in Hanoi, Vietnam. *Journal of Multidisciplinary Healthcare* 2019; 12: 601-6.
86. Nishimura A, Harashima S, Hosoda K, Arai H, Inagaki N. Sex-related differences in frailty factors in older persons with type 2 diabetes: a cross-sectional study. *Ther Adv Endocrinol Metab* 2019; 10: 14.
87. Orkaby AR, Lunetta KL, Sun FJ, et al. Cross-Sectional Association of Frailty and Arterial Stiffness in Community-Dwelling Older Adults: The Framingham Heart Study. *The journals of gerontology Series A, Biological sciences and medical sciences* 2019; 74(3): 373-9.
88. Ottenbacher KJ, Graham JE, Al Snih S, et al. Mexican Americans and frailty: findings from the Hispanic established populations epidemiologic studies of the elderly. *American journal of public health* 2009; 99(4): 673-9.
89. Pilotto A, Noale M, Maggi S, et al. Hypoglycemia Is Independently Associated with Multidimensional Impairment in Elderly Diabetic Patients. *Biomed Res Int* 2014: 7.
90. Pollack LR, Litwack-Harrison S, Cawthon PM, et al. Patterns and Predictors of Frailty Transitions in Older Men: The Osteoporotic Fractures in Men Study. *Journal of the American Geriatrics Society* 2017; 65(11): 2473-9.

91. Presley CA, Chipman J, Min JY, et al. Evaluation of Frailty as an Unmeasured Confounder in Observational Studies of Antidiabetic Medications. *J Gerontol Ser A-Biol Sci Med Sci* 2019; **74**(8): 1282-8.
92. Raji MA, Al Snih S, Ostir GV, Markides KS, Ottenbacher KJ. Cognitive status and future risk of frailty in older Mexican Americans. *Journals of Gerontology Series A: Biomedical Sciences and Medical Sciences* 2010; **65**(11): 1228-34.
93. Ricci NA, Pessoa GS, Ferriolli E, Dias RC, Perracini MR. Frailty and cardiovascular risk in community-dwelling elderly: a population-based study. *Clinical interventions in aging* 2014; 9: 1677-85.
94. Saum KU, Müller H, Stegmaier C, Hauer K, Raum E, Brenner H. Development and Evaluation of a Modification of the F ried Frailty Criteria Using Population-Independent Cutpoints. *Journal of the American Geriatrics Society* 2012; **60**(11): 2110-5.
95. Simpson SH, Lin M, Eurich DT. Medication Adherence Affects Risk of New Diabetes Complications: A Cohort Study. *The Annals of pharmacotherapy* 2016; 50(9): 741-6.
96. Sirola J, Pitkala KH, Tilvis RS, Miettinen TA, Strandberg TE. Definition of frailty in older men according to questionnaire data (RAND-36/SF-36): The Helsinki Businessmen Study. *The journal of nutrition, health & aging* 2011; 15(9): 783-7.
97. Tamura Y, Ishikawa J, Fujiwara Y, et al. Prevalence of frailty, cognitive impairment, and sarcopenia in outpatients with cardiometabolic disease in a frailty clinic. *BMC geriatrics* 2018; 18(1): 264.
98. Tang Z, Wang C, Song X, et al. Co-occurrence of cardiometabolic diseases and frailty in older Chinese adults in the Beijing Longitudinal Study of Ageing. *Age and ageing* 2013; 42(3): 346-51.
99. Tepper S, Alter Sivashensky A, Rivkah Shahar D, Geva D, Cukierman-Yaffe T. The Association between Mediterranean Diet and the Risk of Falls and Physical Function Indices in Older Type 2 Diabetic People Varies by Age. *Nutrients* 2018; 10(6).
100. Thein FS, Li Y, Nyunt MSZ, Gao Q, Wee SL, Ng TP. Physical frailty and cognitive impairment is associated with diabetes and adversely impact functional status and mortality. *Postgraduate medicine* 2018; 130(6): 561-7.
101. Tuttle LJ, Bittel DC, Bittel AJ, Sinacore DR. Early-Onset Physical Frailty in Adults With Diabesity and Peripheral Neuropathy. *Canadian journal of diabetes* 2018; 42(5): 478-83.
102. Vaingankar JA, Chong SA, Abdin E, et al. Prevalence of frailty and its association with sociodemographic and clinical characteristics, and resource utilization in a population of Singaporean older adults. *Geriatr Gerontol Int* 2017; 17(10): 1444-54.
103. van Hateren KJJ, Hendriks SH, Groenier KH, et al. Frailty and the relationship between blood pressure and mortality in elderly patients with type 2 diabetes (Zwolle Outpatient Diabetes project Integrating Available Care-34). *Journal of hypertension* 2015; 33(6): 1162-6.
104. Vaz Fragoso CA, Gahbauer EA, Van Ness PH, Gill TM. Sleep–wake disturbances and frailty in community-living older persons. *Journal of the American Geriatrics Society* 2009; 57(11): 2094-100.
105. Veronese N, Sigeirsdottir K, Eiriksdottir G, et al. Frailty and Risk of Cardiovascular Diseases in Older Persons: The Age, Gene/Environment Susceptibility-Reykjavik Study. *Rejuvenation research* 2017; 20(6): 517-24.
106. Veronese N, Stubbs B, Fontana L, et al. Frailty Is Associated with an Increased Risk of Incident Type 2 Diabetes in the Elderly. *Journal of the American Medical Directors Association* 2016; **17**(10): 902-7.
107. Wang C-P, Lorenzo C, Espinoza SE. Frailty attenuates the impact of metformin on reducing mortality in older adults with type 2 diabetes. *Journal of endocrinology, diabetes & obesity* 2014; **2**(2).

108. Wang C-P, Lorenzo C, Habib SL, Jo B, Espinoza SE. Differential effects of metformin on age related comorbidities in older men with type 2 diabetes. *Journal of diabetes and its complications* 2017; 31(4): 679-86.
109. Watanabe Y, Hirano H, Arai H, et al. Relationship Between Frailty and Oral Function in Community-Dwelling Elderly Adults. *Journal of the American Geriatrics Society* 2017; 65(1): 66-76.
110. Weinstein G, Lutski M, Goldbourt U, Tanne D. Physical frailty and cognitive function among men with cardiovascular disease. *Archives of gerontology and geriatrics* 2018; 78: 1-6.
111. Wong CH, Weiss D, Sourial N, et al. Frailty and its association with disability and comorbidity in a community-dwelling sample of seniors in Montreal: a cross-sectional study. *Aging Clin Exp Res* 2010; 22(1): 54-62.
112. Wong TY, Sofia Massa M, O'Halloran AM, Kenny RA, Clarke R. Cardiovascular risk factors and frailty in a cross-sectional study of older people: Implications for prevention. *Age and Ageing* 2018; 47(5): 714-20.
113. Woo J, Yang X, Lui LT, et al. Utility of the FRAIL Questionnaire in Detecting Heart Failure with Preserved Ejection Fraction. *Journal of Nutrition, Health and Aging* 2019; 23(4): 373-7.
114. Wu C, Smit E, Xue Q-L, Odden MC. Prevalence and correlates of frailty among community-dwelling Chinese older adults: the China Health and Retirement Longitudinal Study. *The Journals of Gerontology: Series A* 2018; 73(1): 102-8.
115. Wu IC, Shiesh S-C, Kuo P-H, Lin X-Z. High oxidative stress is correlated with frailty in elderly chinese. *Journal of the American Geriatrics Society* 2009; 57(9): 1666-71.
116. Xue Q, Qin MZ, Jia J, Liu JP, Wang Y. Association between frailty and the cardio-ankle vascular index. *Clinical Interventions in Aging* 2019; 14: 735-42.
117. Yanagita I, Fujihara Y, Eda T, et al. Low glycated hemoglobin level is associated with severity of frailty in Japanese elderly diabetes patients. *Journal of diabetes investigation* 2018; 9(2): 419-25.
118. Zaslavsky O, Walker RL, Crane PK, Gray SL, Larson EB. Glucose Levels and Risk of Frailty. *The journals of gerontology Series A, Biological sciences and medical sciences* 2016; **71**(9): 1223-9.
